# Explainable Ensemble Boosting Framework for Chronic Disease Risk Prediction Using Lifestyle and Clinical Data: A Multi-Dataset Study

**DOI:** 10.3390/life16101734

**Published:** 2026-10-15

**Authors:** Shahid Mohammad Ganie

**Affiliations:** Department of Health Information Management and Technology, College of Applied Medical Sciences, King Faisal University, Al-Ahsa 31982, Saudi Arabia; sganie@kfu.edu.sa

**Keywords:** chronic disease prediction, ensemble learning, boosting algorithms, explainable artificial intelligence, SHAP, LIME, probability calibration, repeated cross-validation

## Abstract

Chronic diseases represent a substantial public health burden, motivating the development of transparent and reproducible approaches for disease-specific risk prediction using routinely available lifestyle and clinical variables. This study presents a common explainable ensemble boosting framework for comparative prediction across three independent disease-specific datasets: heart disease, lung cancer, and diabetes. Five boosting algorithms, including XGBoost, CatBoost, LightGBM, AdaBoost, and Gradient Boosting, were evaluated under a consistent leakage-free protocol. Each dataset was first split into stratified 80% training and 20% held-out test sets, with preprocessing, random oversampling, hyperparameter optimization, probability calibration, and threshold selection performed only on the training data. Performance was assessed using accuracy, balanced accuracy, precision, sensitivity, specificity, F1-score, ROC-AUC, PR-AUC, Brier score, and bootstrap confidence intervals. On the held-out test sets, AdaBoost achieved the highest balanced accuracy (68.07%) and ROC-AUC (71.71%) for heart disease; CatBoost achieved the highest balanced accuracy (92.59%) and ROC-AUC (96.30%) for lung cancer, although these estimates require caution because the lung-cancer test set contained only eight negative observations; and XGBoost achieved the highest balanced accuracy (77.02%), whereas Gradient Boosting achieved the highest ROC-AUC (82.43%) for diabetes. Repeated stratified cross-validation showed significant differences among the five algorithms for balanced accuracy (Friedman $\chi^{2}=26.293$, p<0.001), ROC-AUC ($\chi^{2}=21.173$, p<0.001), and F1-score ($\chi^{2}=15.093$, p=0.0045). Across these endpoints, Gradient Boosting achieved the best mean rank (1.98), followed by CatBoost (2.47), although no model was uniformly the highest-performing across all datasets and evaluation measures. SHAP and LIME analyses were additionally used to characterize global and local feature contributions to model predictions. The findings demonstrate the feasibility of a common explainable boosting workflow across heterogeneous disease-specific benchmark datasets while emphasizing that these results constitute internal benchmark evidence and require independent external and prospective validation before clinical application.

## 1. Introduction

The global burden of chronic diseases has increased substantially over the past decade, with a growing proportion of individuals experiencing multiple chronic conditions simultaneously [1]. This phenomenon, commonly referred to as multi-chronic diseases (MCDs), is characterized by long-term persistence, shared risk factors, and complex disease interactions, placing significant strain on individuals, healthcare systems, and societies [2]. The rapid increase in the number of patients suffering from these diseases has become a major global concern [3]. Unlike single-disease conditions, MCDs often arise from shared lifestyle, behavioral, and physiological risk factors, making early identification and integrated risk assessment critically important for preventive care and personalized intervention strategies. Lifestyle-related disorders have been widely recognized as major contributors to the onset and progression of chronic cardiometabolic and metabolic conditions, including hypertension, diabetes, obesity, and cardiovascular disease [4]. Despite this well-established association, most existing clinical screening and diagnostic approaches remain disease-specific, focusing on isolated outcomes rather than capturing the cumulative and interacting risks of multiple chronic conditions driven by shared lifestyle and clinical factors [5].

According to the World Health Organization (WHO) (https://www.who.int/news-room/fact-sheets/detail/noncommunicable-diseases accessed on 21 June 2026), noncommunicable diseases caused at least 43 million deaths in 2021, accounting for approximately 75% of non-pandemic-related deaths worldwide. Cardiovascular diseases (https://www.afro.who.int/health-topics/cardiovascular-diseases accessed on 22 June 2026) accounted for the largest share of NCD mortality, followed by cancers, chronic respiratory diseases, and diabetes [6]. Similarly, the International Diabetes Federation (IDF) (https://diabetesatlas.org/resources/previous-editions/ accessed on 28 June 2026) reported that in 2021, around 537 million adults (aged 20–79 years) had diabetes. It has been estimated that the number of people living with diabetes will reach an alarming 643 million by 2030 and will increase by an average of 10 million people per year over a period of 15 years, finally reaching about 783 million diabetics by 2045. Lung cancer is the leading cancer globally, and one in every 16 people will be diagnosed with the global incidence of lung cancer in 2022 (https://www.iarc.who.int/ accessed on 2 July 2026). With so many millions of new cases and fatalities each year, lung cancer continues to be the most prevalent and the leading cause of an increase in the number of cancer patients and mortality [7]. These conditions share several modifiable behavioral and metabolic determinants, including tobacco use, physical inactivity, unhealthy diet, elevated blood pressure, abnormal blood glucose, and excess body weight. Their substantial global burden underscores the importance of transparent and reproducible approaches for disease-specific risk prediction and early identification using routinely available health information.

Early identification of chronic disease risk can enable individuals to adopt positive lifestyle changes related to diet, physical activity, and health behaviors, potentially delaying disease onset and reducing long-term complications [8,9,10]. In recent years, the growing availability of healthcare and lifestyle data, generated from sources such as sensors [11], health databases [12], social and digital platforms [13], wearable devices [14], and structured clinical pathways have created new opportunities for computational risk modeling. Such data, combined with demographic and lifestyle indicators (e.g., age, gender, body composition, smoking and alcohol habits), have been widely used to support predictive modeling for chronic conditions, including diabetes, cardiovascular disease, liver disorders, and kidney disease [15,16].

**Figure 1:**
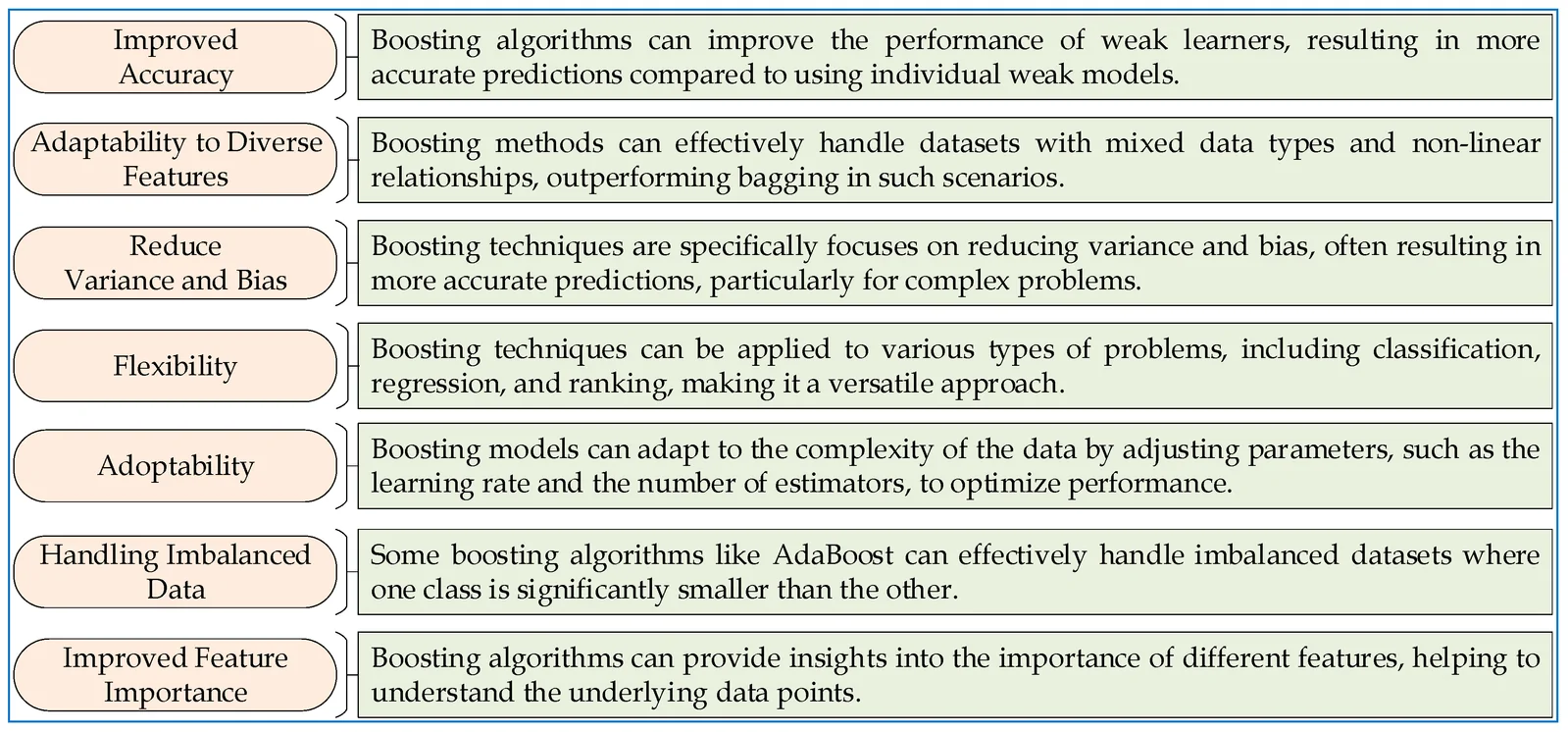
Advantages of boosting techniques.

Within this context, machine learning (ML) techniques, including ensemble learning (EL) methods, have gained increasing attention for disease risk prediction and diagnostic support [17]. These methods are capable of extracting complex, nonlinear patterns from high-dimensional data and have demonstrated improved predictive stability compared to single-model approaches [18,19]. By leveraging mathematical optimization and data-driven learning, ML-based models can support analytical risk assessment while reducing the cognitive burden on healthcare practitioners [20,21,22]. Ensemble learning, in particular, combines multiple learners to improve robustness and generalization, offering advantages over traditional single-model approaches [23]. Figure 1 highlights key advantages of boosting techniques.

Despite advances in machine learning for chronic disease prediction, several methodological challenges remain. Many studies evaluate individual diseases using dataset-specific preprocessing, model selection, and validation procedures, making it difficult to determine whether differences in reported performance reflect algorithmic behavior or experimental design. Interpretability is another important consideration because high predictive performance alone does not explain how individual variables influence model outputs. In addition, inadequate separation of training and test data during preprocessing or class balancing can lead to overly optimistic performance estimates, while discrimination measures alone do not indicate whether predicted probabilities are adequately calibrated. These considerations motivate comparative evaluation frameworks that emphasize methodological consistency, leakage prevention, multiple complementary performance measures, probability calibration, statistical comparison, and model explainability.

Accordingly, this study presents a unified experimental framework for benchmarking five boosting algorithms, XGBoost, CatBoost, LightGBM, AdaBoost, and Gradient Boosting—across three independent chronic disease prediction tasks: heart disease, lung cancer, and diabetes. Because the datasets represent independent populations and do not contain shared patient identifiers, each disease is modeled separately as a binary classification task, and no patient-level multimorbidity or aggregated multi-disease risk score is estimated. This distinction enables the study to evaluate the behavior, robustness, calibration, and interpretability of the same family of boosting algorithms across heterogeneous disease-specific datasets without implying clinical integration that the available data cannot support. The main contributions of this study are as follows:

1. A multi-dataset benchmarking framework is applied for evaluating five boosting algorithms across independent heart disease, lung cancer, and diabetes prediction tasks using the same overarching experimental principles.
2. A leakage-free evaluation protocol is implemented in which the original data are partitioned into stratified training and held-out test sets before fitted preprocessing or class balancing, while preprocessing, random oversampling, hyperparameter optimization, probability calibration, and threshold determination are restricted to the training data.
3. Model performance is evaluated using complementary discrimination, classification, and calibration measures, including accuracy, balanced accuracy, precision, sensitivity, specificity, F1-score, ROC-AUC, PR-AUC, Brier score, and bootstrap confidence intervals, thereby avoiding conclusions based solely on overall accuracy.
4. Cross-model differences are examined using repeated stratified cross-validation and matched non-parametric statistical testing. Friedman tests are followed, when significant, by pairwise Wilcoxon signed-rank tests with Holm adjustment, providing a more rigorous assessment of whether numerical differences between boosting algorithms are consistently observed across the evaluated tasks.
5. Global and local model behavior is examined using SHAP and LIME within the leakage-free experimental framework. The resulting explanations are interpreted as feature contributions to model predictions rather than evidence of causal clinical relationships

The remainder of this paper is organized as follows. Section 2 reviews studies on machine-learning- and ensemble-based chronic disease prediction and identifies methodological gaps addressed here. Section 3 presents the methodology, including the leakage-free workflow, boosting algorithms, hyperparameter optimization, probability calibration, threshold selection, performance evaluation, repeated cross-validation, statistical analysis, and SHAP- and LIME-based explainability. Section 4 describes the three independent disease-specific datasets and their characteristics. Section 5 presents exploratory data analysis, leakage-free preprocessing, and training-fold handling of class imbalance. Section 6 reports held-out test performance, calibration, repeated cross-validation, statistical comparisons, and disease-specific SHAP and LIME analyses. Section 7 discusses findings, limitations, and clinical implications. Section 8 compares the evaluated boosting algorithms within existing studies. Finally, Section 9 summarizes the conclusions and future research directions, while Appendix A provides detailed dataset characteristics and supplementary exploratory analyses.

## 2. Related Work

Recent advances in machine learning have substantially expanded data-driven prediction of chronic diseases using demographic, lifestyle, physiological, and clinical variables. Previous studies have investigated cardiovascular disease, diabetes, cancer, and other chronic conditions using conventional machine learning, ensemble learning, and deep learning approaches. Although these studies demonstrate the predictive potential of computational models, direct comparison of reported performance remains challenging because studies frequently differ in dataset characteristics, preprocessing procedures, class-balancing strategies, model-selection protocols, evaluation metrics, and validation designs. Consequently, there is value in examining established learning algorithms within a consistent experimental framework across heterogeneous, disease-specific datasets, while maintaining independent prediction tasks for each disease.

Ramadhan et al. [24] presented a comprehensive systematic literature review on chronic disease prediction using machine learning, emphasizing the critical role of data preprocessing in improving predictive performance. The study systematically analyzed challenges such as missing values, feature selection, class imbalance, outliers, and normalization across major chronic conditions and highlighted ensemble and deep learning methods as dominant approaches while identifying open research gaps in preprocessing-aware model design. For example, Abdalrada et al. [25] proposed a two-stage model for diabetes and cardiovascular diseases, achieving 94.09% accuracy, 93.5% sensitivity, and 95.8% specificity on a dataset of 2000 records. Similarly, Hossain et al. [26] evaluated six ML models on clinical datasets comprising 4819 samples for type 2 diabetes (T2D) and 5731 samples for cardiovascular disease (CVD), reporting accuracies ranging from 79% to 88%. In another study, Chen et al. [27] proposed a convolutional neural network-based multimodal disease risk prediction framework (CNN-MDRP) for predicting disease risk from heterogeneous data. This approach considers all data formats for risk prediction. Notably, the proposed algorithm achieves 94.8% prediction accuracy, outperforming several conventional prediction algorithms. Gupta and Singh [28] tested multiple chronic disease prediction frameworks, with AdaBoost achieving 91.90% accuracy on the Cleveland dataset and 96.60% on the Pima dataset. Also, Arumugam et al. [29] evaluated multiple disease prediction models using a decision tree (DT), Naive Bayes (NB), and a support vector machine (SVM), reporting accuracies of 90%, 78%, and 86%, respectively. They applied hyperparameter tuning to achieve optimal results. The authors further discussed the potential application of these models in healthcare decision support. Sandhiya and Palani [30] incorporated incremental feature selection and CNNs for predicting heart disease, diabetes, and cancer.

In addition, Alanazi [31] combined CNNs for automated feature extraction with KNN for prediction, and Raheja et al. [32] developed a multi-disease framework using logistic regression, random forest, KNN, decision tree, and CNN, validated through multiple performance metrics. In other studies, Lee et al. [33] developed a high-performance prediction model for four chronic diseases using ML techniques. The models include logistic regression, gradient boosting machine, random forest, and XGBoost. Among all the algorithms, XGBoost achieved the highest performance, with AUC scores of 0.9303 for type 2 diabetes, 0.8704 for hypertension, 0.8442 for hyperlipidemia, and 0.8726 for cardiovascular disease. These scores indicate the XGBoost model’s effectiveness in predicting disease occurrence, with higher scores indicating better performance. Kim et al. [34] advanced this work using a CNN-LSTM multitask framework with time-series data, effectively capturing feature correlations across multiple chronic diseases. The CNN model was employed to extract local features for each task, while the long short-term memory (LSTM) algorithm captured feature variations and extracted temporal features for model building. Finally, the CNN-LSTM model explored shared LSTM blocks to facilitate multi-task learning to improve overall performance.

Furthermore, Kumar et al. [35] developed an efficient real-time multi-level chronic disease prediction and recommendation model (RMCDPRM) system. Deep neural networks are integrated with feature-depth clustering to handle the large-scale, heterogeneous medical data used in this work. The proposed model estimates class-level disease support and disease weight for multi-stage disease prediction and then applies a recommendation mechanism for hospitals and practitioners. The experimental results demonstrate high prediction accuracy (up to 99%) and reduced time complexity compared to conventional deep learning and machine learning methods. Mulakala et al. [36] proposed a model using machine learning and deep learning techniques for multiple diseases, including diabetes, heart disease, lung cancer, and brain tumors. The study employs classical ML models (e.g., Random Forest, SVM) and CNN-based architectures for image-based diagnosis, achieving high predictive accuracy across diseases. The authors suggested that the proposed model lacks model interpretability and a unified disease-level representation. Yew et al. [37] proposed the XGBoost model to investigate multiple chronic condition (MCC) patterns among individuals with Alzheimer’s disease and related dementia. The model achieved an overall AUROC of 0.71 by exploring social, mental, physical, and dementia-related factors. While the approach captures complex disease interactions, it primarily focuses on structured clinical variables and does not address explainability at the model decision level.

Recent studies emphasize the need for interpretable and generalizable machine-learning frameworks in healthcare. Nemane et al. [38] reviewed federated learning in multimodal healthcare and identified data heterogeneity, class imbalance, privacy, fairness, and interpretability as challenges in AI systems. Mahmud et al. [39] proposed an explainable stacking ensemble for cardiovascular disease risk prediction that combines clinically engineered features, multiple classifiers, SHAP-based interpretation, statistical testing, and external validation. Their findings reflect an emphasis on performance, interpretability, and rigorous validation. Nevertheless, the literature remains heterogeneous in datasets, preprocessing, handling of imbalance, model selection, validation design, and evaluation measures, limiting comparisons across studies.

Collectively, the literature demonstrates substantial progress in machine-learning-based chronic disease prediction, but several methodological gaps remain relevant to comparative benchmarking. First, preprocessing and class-balancing procedures are not always clearly separated from model evaluation, making rigorous leakage prevention an important consideration when estimating generalization performance. Second, comparisons across algorithms are frequently based primarily on accuracy or a limited set of discrimination metrics, whereas imbalanced healthcare datasets require complementary measures such as balanced accuracy, sensitivity, specificity, F1-score, ROC-AUC, and PR-AUC. Third, probability calibration is less frequently examined despite its importance when model outputs are interpreted as risk estimates. Fourth, numerical differences among competing algorithms are not always accompanied by matched statistical comparisons that account for repeated model evaluation. Finally, although SHAP, LIME, and related explainability techniques are increasingly used in healthcare machine learning, their integration with leakage-aware validation, calibration assessment, and systematic cross-model comparison remains an important methodological consideration. These gaps motivate the present study, which applies a common experimental protocol to five boosting algorithms across three independent disease-specific datasets while preserving separate prediction tasks for heart disease, lung cancer, and diabetes.

## 3. Research Methodology

The proposed framework provides a common experimental protocol for evaluating five boosting algorithms across three independent binary classification tasks involving heart disease, lung cancer, and diabetes. Each dataset was analyzed independently; therefore, predictions from different disease datasets were not aggregated into a common patient-level risk score. The workflow was specifically designed to maintain strict separation between model development and final evaluation and to prevent information from the held-out test set from influencing preprocessing, class balancing, hyperparameter optimization, probability calibration, or decision-threshold selection.

### 3.1. Experimental Design Workflow

**Figure 2:**
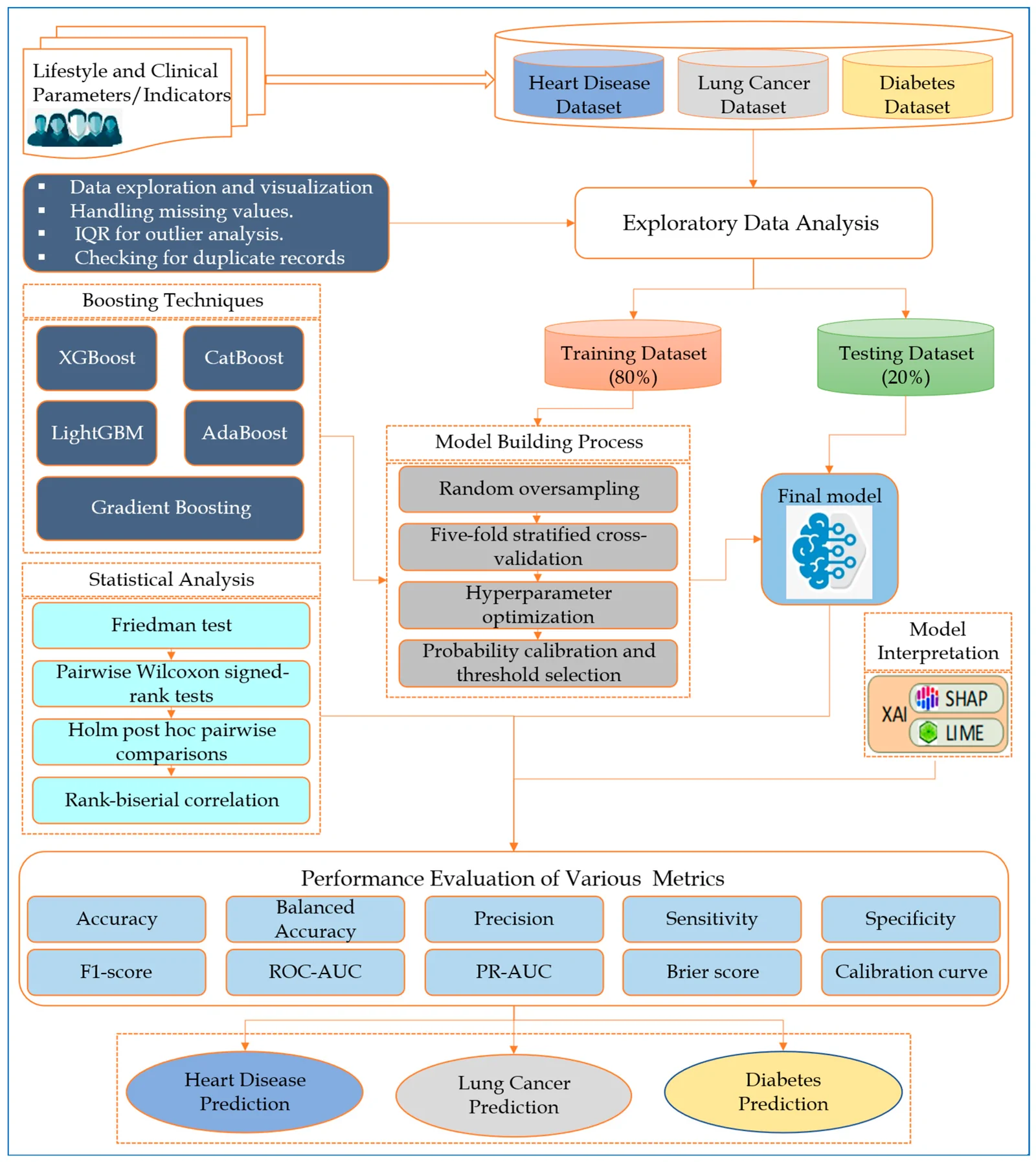
Proposed methodology for comparative boosting-based prediction across the heart disease, lung cancer, and diabetes datasets.

As illustrated in Figure 2, the experimental workflow comprised the following principal components: stratified train–test partitioning, training-only preprocessing and class-imbalance handling, hyperparameter optimization using stratified cross-validation, probability calibration and decision-threshold selection, final evaluation on the untouched held-out test set, repeated cross-validation and statistical assessment, and explainability analysis.

- **Dataset Partitioning:** Each disease-specific dataset was partitioned into training and test subsets using a stratified 80:20 split to preserve class proportions before preprocessing or balancing. The test subset was held out from development, hyperparameter selection, preprocessing parameter fitting, oversampling, calibration, and decision threshold optimization.
- **Training-Only Preprocessing:** All preprocessing was fitted on training data and applied without re-estimation. Missing values were handled using training-data statistics. In the diabetes dataset, implausible zeros in Glucose, Blood Pressure, Skin Thickness, Insulin, and Body Mass Index were treated as missing and median-imputed in the training pipeline; zeros for Pregnancies were retained as valid, so non-training observations did not directly contribute to parameter estimation.
- **Outlier Assessment:** Outliers were examined using the interquartile range (IQR). Observations were not removed for exceeding IQR-based limits because extreme values may reflect variation, and removal could alter the data distribution.
- **Class imbalance:** Random oversampling was used only within training, never before the train–test split. During cross-validation, RandomOverSampler was applied to the training folds, while the validation folds retained the original distribution. The test set was never resampled, preventing duplicates across partitions. Oversampling was preferred because synthetic interpolation could yield implausible combinations of binary, categorical, and continuous variables.
- **Boosting models:** Five classifiers, including XGBoost, CatBoost, LightGBM, AdaBoost, and Gradient Boosting, were evaluated. The optimization criterion was dataset-specific: mean cross-validated F1-score was used for heart disease, whereas mean cross-validated balanced accuracy was used for lung cancer and diabetes.
- **Probability calibration:** It was incorporated because discrimination alone does not ensure probabilities. Calibration used training data: out-of-fold predictions produced calibrated probabilities and thresholds, selected from training-derived predictions, not the test set. The calibrated model and threshold were applied to observations. Quality was assessed using the Brier score and reliability curves.
- **Held-Out Test Evaluation:** After model selection, calibration, and training-derived threshold determination, each model was evaluated on the test set. No test labels were used during preprocessing, resampling, hyperparameter optimization, calibration, or threshold selection. Performance was assessed using accuracy, balanced accuracy, precision, sensitivity (recall), specificity, F1-score, ROC-AUC, PR-AUC, and Brier score. Confusion matrices characterized behavior.
- **Repeated Cross-Validation and Statistical Assessment:** Stratified cross-validation used five folds and five repetitions across five boosting algorithms to assess stability beyond the train–test partition. Balanced accuracy was primary; ROC-AUC and F1-score were secondary. Results averaged by repetition. Differences assessed by Friedman test, Holm-adjusted Wilcoxon signed-rank tests, and rank-biserial correlation, providing evidence, not external validation.
- **Explainability Analysis:** Explainability analyses were conducted after development to characterize global and local behavior. SHAP quantified contributions and influence across observations, whereas LIME explained test instances locally. Explanations described model behavior rather than causal clinical relationships. Representative cases illustrated patterns without implying certainty.

### 3.2. Boosting Methods

Ensemble learning techniques have been widely applied to complex prediction problems across multiple domains [38,39,40]. In healthcare, their application to disease detection, diagnosis, and prognosis has expanded considerably, with increasing emphasis on robust predictive modeling and systematic comparative evaluation [41]. These algorithms represent different implementations of sequential ensemble learning and were selected to enable a consistent comparison of widely used boosting strategies under the same leakage-free experimental protocol. The same model-development principles were applied to the heart disease, lung cancer, and diabetes tasks, while hyperparameter optimization was conducted independently for each dataset. The considered boosting techniques are discussed below:

1. XGBoost: Extreme Gradient Boosting (XGBoost) is an optimized implementation of gradient-boosted decision trees designed for computational efficiency and predictive performance. It incorporates parallel processing, regularization, and efficient handling of missing values to improve scalability and reduce overfitting. XGBoost can also provide model-based feature-importance measures that characterize the relative contribution of predictors to the fitted model [42].
2. CatBoost: CatBoost (Categorical Boosting) is a gradient-boosting algorithm designed to efficiently handle categorical features while reducing the need for extensive manual encoding. It incorporates specialized strategies for processing categorical variables and controlling prediction bias during model training. These characteristics make CatBoost suitable for datasets containing heterogeneous predictor types [43].
3. LightGBM: Light Gradient Boosting Machine (LightGBM) is an efficient gradient-boosting framework designed for computationally scalable model training. It employs histogram-based learning and leaf-wise tree growth and incorporates techniques such as Gradient-based One-Side Sampling to improve training efficiency. These characteristics enable LightGBM to handle relatively large and high-dimensional datasets with reduced computational requirements [44].
4. AdaBoost: Adaptive Boosting (AdaBoost) is a sequential ensemble method that combines multiple weak learners to construct a stronger predictive model. During iterative training, greater weight is assigned to observations that were misclassified by preceding learners, thereby directing subsequent learners toward more difficult cases. The final prediction is obtained by combining the weighted contributions of the individual weak learners. AdaBoost is widely applied to both classification and regression tasks [45].
5. Gradient Boosting: Gradient Boosting constructs an ensemble sequentially by fitting each new weak learner to reduce the residual errors of the existing ensemble. At each iteration, the algorithm minimizes a specified loss function using gradient-based optimization, and the newly fitted learner is added to the accumulated model. This iterative procedure enables progressive refinement of predictions and is widely used for classification and regression tasks [46].

### 3.3. Hyperparameter Tuning

Hyperparameter optimization was conducted independently for each boosting algorithm and disease-specific dataset, using only the training partition. Grid search with five-fold stratified cross-validation was employed, with shuffling enabled and a fixed random seed to support reproducibility. Preprocessing and random oversampling were incorporated within the model pipeline so that these operations were fitted independently within each cross-validation training fold, thereby preventing information from the corresponding validation fold from entering model development. The held-out test set was not involved in hyperparameter optimization.

The optimization criterion was selected according to the characteristics of each prediction task. For the heart disease dataset, the mean cross-validated F1-score was used because the positive heart disease class was substantially underrepresented, and the F1-score jointly reflects precision and sensitivity for the positive class. For the lung cancer and diabetes datasets, balanced accuracy was used as the optimization criterion to account for class imbalance by giving equal weight to sensitivity and specificity. The hyperparameter configuration yielding the highest mean cross-validation score was selected separately for each model and dataset. Table 1 summarizes the search spaces used in the experiments.

### 3.4. Probability Calibration and Decision-Threshold Selection

Following hyperparameter optimization, probability calibration was performed using only the training data. Each tuned classifier was wrapped with sigmoid calibration via CalibratedClassifierCV. Fivefold stratified calibration was used for the heart disease and diabetes datasets, whereas threefold stratified calibration was used for the lung cancer dataset due to its smaller minority class. The held-out test set was not used during calibration.

Because a fixed probability threshold of 0.50 may not provide an appropriate balance between sensitivity and specificity for imbalanced clinical datasets, model-specific classification thresholds were derived exclusively from the training data. For each tuned model, out-of-fold calibrated probabilities were generated using five-fold stratified cross-validation. The optimal threshold was selected using Youden’s J statistic, defined as J=Sensitivity + Specificity−1, which identifies the threshold that maximizes the separation between the true-positive rate and false-positive rate. The resulting threshold was then locked before evaluating the held-out test set. Consequently, neither the calibration parameters nor the classification thresholds were estimated using test-set labels.

### 3.5. Evaluation Metrics

Model performance was evaluated on the untouched held-out test set using complementary measures of classification, discrimination, and calibration. The reported metrics included accuracy, balanced accuracy, precision, sensitivity, specificity, F1-score, area under the receiver operating characteristic (ROC-AUC) curve, area under the precision–recall (PR-AUC) curve, and Brier score. Confusion matrices were additionally generated to report true-negative, false-positive, false-negative, and true-positive predictions. ROC-AUC and PR-AUC were calculated from predicted probabilities rather than hard class assignments.

Accuracy represents the proportion of all observations correctly classified, whereas balanced accuracy is the average of sensitivity and specificity and was included because the class distributions were unequal. Precision quantifies the proportion of predicted positive observations that are true positives, while sensitivity measures the proportion of actual positive cases that are correctly identified. Specificity measures the proportion of actual negative cases correctly classified. The F1-score represents the harmonic mean of precision and sensitivity. ROC-AUC evaluates the model’s discriminative power across classification thresholds, whereas PR-AUC emphasizes the precision–recall trade-off and is particularly informative when class frequencies are imbalanced. The Brier score measures the mean squared difference between predicted probabilities and observed binary outcomes; lower values indicate better probabilistic accuracy and calibration.

To quantify uncertainty in the held-out results, percentile-based bootstrap 95% confidence intervals were generated after model fitting and threshold selection. Bootstrap resampling was applied only to the final held-out predictions and therefore did not influence training, calibration, model selection, or threshold determination. The final notebooks calculate confidence intervals for accuracy, balanced accuracy, precision, sensitivity, specificity, F1-score, ROC-AUC, PR-AUC, and Brier score.

Evaluating the performance of machine learning and ensemble models is critical to understanding their efficacy and suitability for specific tasks. The choice of evaluation metrics depends on the prediction task and the underlying class distribution. In this study, model performance was evaluated separately for each disease-specific binary classification task. Let TP, TN, FP, and FN denote true positives, true negatives, false positives, and false negatives, respectively.

### 3.6. Cross-Validation and Statistical Analysis

In addition to the held-out test evaluation, repeated stratified cross-validation was conducted to assess the relative stability of the five boosting algorithms. For each disease dataset, five-fold stratified cross-validation was repeated five times using matched splits across models, producing 25-fold-level evaluations per algorithm. The same model-specific configurations established during the development experiment were used for this supplementary comparative analysis.

Because the five folds within the same repetition are not statistically independent, the fold-level results were first averaged within each repetition. This produced one performance value per model for each dataset–repeat combination. Across the three datasets and five repetitions, 15 matched dataset–repeat blocks were therefore available for statistical comparison. Balanced accuracy was defined as the primary comparative endpoint, while ROC-AUC and F1-score were evaluated as secondary endpoints.

For each endpoint, the non-parametric Friedman test was first applied to examine whether overall performance differed among XGBoost, CatBoost, LightGBM, AdaBoost, and Gradient Boosting across the matched blocks. When the omnibus Friedman test was statistically significant, pairwise Wilcoxon signed-rank tests were performed between all model pairs. The Holm adjustment was applied across the 10 pairwise comparisons to control the family-wise error rate. Rank-biserial correlation was also calculated as an effect size for the paired comparisons. Statistical significance was defined as an adjusted (p<0.05).

Because repeated cross-validation observations remain partially correlated and the analysis is based on only three public datasets, these statistical comparisons were interpreted as supportive evidence of internal comparative performance rather than as proof of the highest-performing model for the specified endpoint or external clinical generalizability.

### 3.7. Explainable AI

Explainable artificial intelligence (XAI) analysis was used to characterize how the trained boosting models used the available features to generate disease-specific predictions. Two complementary approaches, SHapley Additive exPlanations (SHAP) and Local Interpretable Model-Agnostic Explanations (LIME), were employed. SHAP was used to characterize global feature contributions and the direction and magnitude of feature contributions on model outputs, whereas LIME was used to provide local explanations for selected individual observations. The XAI analyses were performed separately for the heart disease, lung cancer, and diabetes prediction tasks.

To avoid selecting an explanation model based on held-out test performance, the XAI model was determined exclusively from training-derived out-of-fold evidence. For heart disease, the model was selected by ranking the candidate algorithms based on out-of-fold ROC-AUC and F1-score at the locked training-derived threshold, with AdaBoost selected for explanation. For lung cancer and diabetes, out-of-fold balanced accuracy, followed by ROC-AUC, was used for model selection, yielding CatBoost and AdaBoost, respectively. The corresponding tuned base classifiers were subsequently refitted on the complete training partitions for explainability analysis.

#### 3.7.1. SHAP-Based Global Explanation

SHAP analysis was applied to the underlying tuned boosting classifier after the preprocessing transformations learned from the training data had been applied. Global feature importance was summarized using the mean absolute SHAP value across the analyzed observations, while SHAP summary visualizations were used to examine the relative contribution of individual predictors to model outputs. The explainer background and reference information were derived from the training data rather than the held-out test set. Because probability calibration modifies the mapping of model scores to predicted probabilities but does not redefine the feature relationships learned by the underlying boosting estimator, the tuned base classifier was used for the feature-attribution analysis.

#### 3.7.2. LIME-Based Local Explanation

LIME was used as a complementary local explanation method to characterize the contribution of individual features to selected predictions. The LIME explainer was constructed from the transformed training distribution, thereby preventing held-out test observations from being used to establish the explainer reference distribution. Representative observations were then explained by locally approximating the behavior of the corresponding fitted classifier around each selected instance. Continuous variables were discretized within the LIME implementation, and a fixed random state was used to improve reproducibility. The final diabetes implementation, for example, constructs LimeTabularExplainer exclusively from the transformed training data and uses the fitted base classifier to obtain class probabilities.

SHAP and LIME outputs were interpreted as explanations of model behavior rather than evidence of causal clinical relationships. Accordingly, a positive or negative feature contribution indicates that a feature shifted the model output toward or away from a predicted class for the analyzed observation; it does not establish that the corresponding variable causes or prevents the disease. This distinction is particularly important because the three datasets are observational benchmark datasets and were not designed for causal inference.

### 3.8. Hardware and Software Specifications

All experiments were implemented in Python 3.10.20 using the Jupyter Notebook environment. The computational workflow incorporated standard machine-learning and data-analysis libraries, including scikit-learn for preprocessing, model selection, calibration, and performance evaluation; imbalanced-learn for training-fold random oversampling; XGBoost, CatBoost, and LightGBM for their respective boosting implementations; and SHAP and LIME for explainability analysis. The experiments were conducted on a workstation equipped with an Intel^®^ Core™ i9-10900K processor (Micro-Star International, New Taipei City, Taiwan) operating at 3.70 GHz, 64 GB DDR4 RAM, a 500 GB NVMe solid-state drive, and a 2 TB hard disk, running Windows 11 Pro. The experimental configuration is summarized in Table 2.

## 4. Dataset Selection and Description

Three publicly available datasets were used to evaluate the proposed framework across independent disease-specific prediction tasks involving heart disease (https://www.kaggle.com/datasets/christofel04/cardiovascular-study-dataset-predict-heart-disea accessed on 10 May 2026), lung cancer (https://www.kaggle.com/datasets/jillanisofttech/lung-cancer-detection accessed on 10 May 2026), and diabetes (https://www.kaggle.com/code/mragpavank/pima-indians-diabetes-database accessed on 10 May 2026). The heart disease dataset contains 3390 observations and 15 variables, comprising 14 demographic, lifestyle, and clinical predictors and one binary outcome variable. The lung cancer dataset contains 309 observations and 16 variables, comprising 15 demographic, lifestyle, and self-reported symptom-related predictors and one binary outcome variable. The diabetes dataset contains 768 observations and nine variables, comprising eight clinical and demographic predictors and one binary outcome variable. A summary of the three datasets is presented in Table 3. Detailed variable definitions, coding schemes, measurement information, observed ranges, and descriptive statistics are provided in Table A1, Table A2, Table A3, Table A4, Table A5 and Table A6 (Appendix A).

## 5. Exploratory Data Analysis and Preprocessing

Exploratory data analysis (EDA) was conducted separately for the heart disease, lung cancer, and diabetes datasets to characterize their distributions, identify potential data-quality issues, examine class imbalance, and understand relationships among the available variables [47]. The exploratory analyses were descriptive and were not used to derive parameters subsequently applied to the held-out test observations. For predictive modeling, all fitted preprocessing and resampling procedures were implemented within the training pipeline after the stratified train–test partition, as described in Section 3. This distinction ensured that descriptive exploration did not result in information leakage during model development or final evaluation.

### 5.1. Exploratory Data Analysis

**Figure 3:**
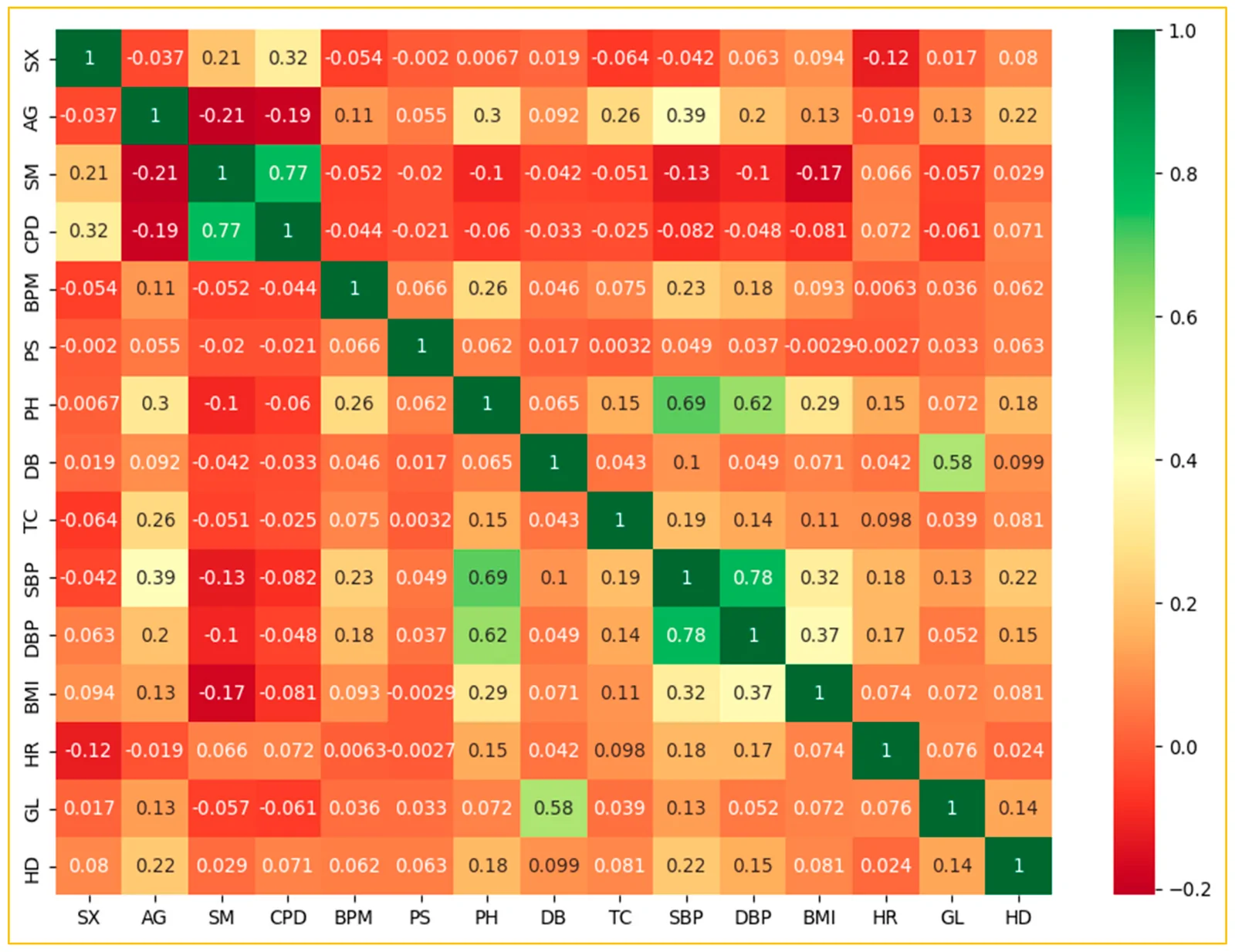
Correlation matrix of numerical variables in the heart disease dataset.

**Figure 4:**
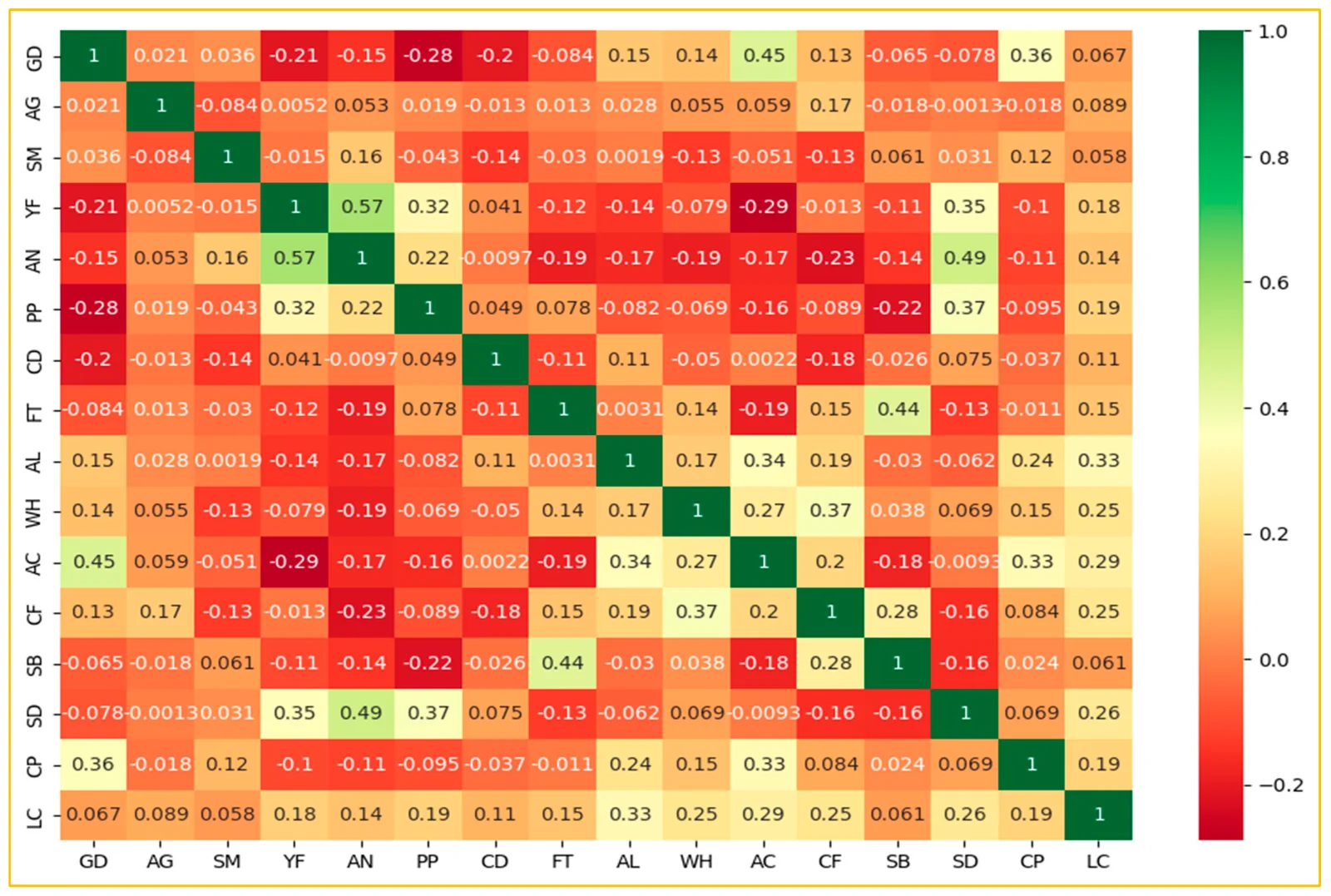
Correlation matrix of encoded variables in the lung cancer dataset.

**Figure 5:**
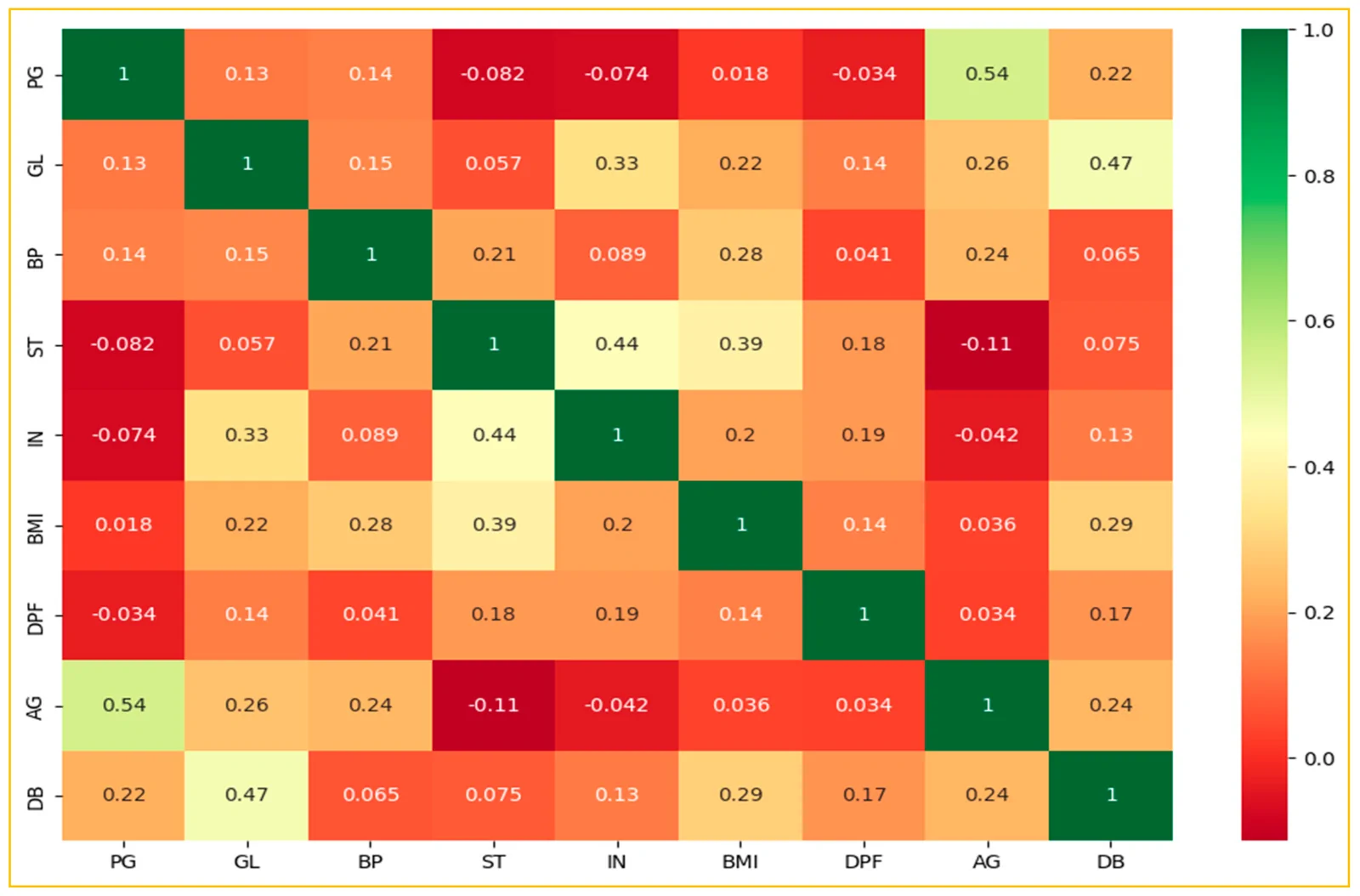
Correlation matrix of numerical variables in the diabetes dataset.

**Figure A1:**
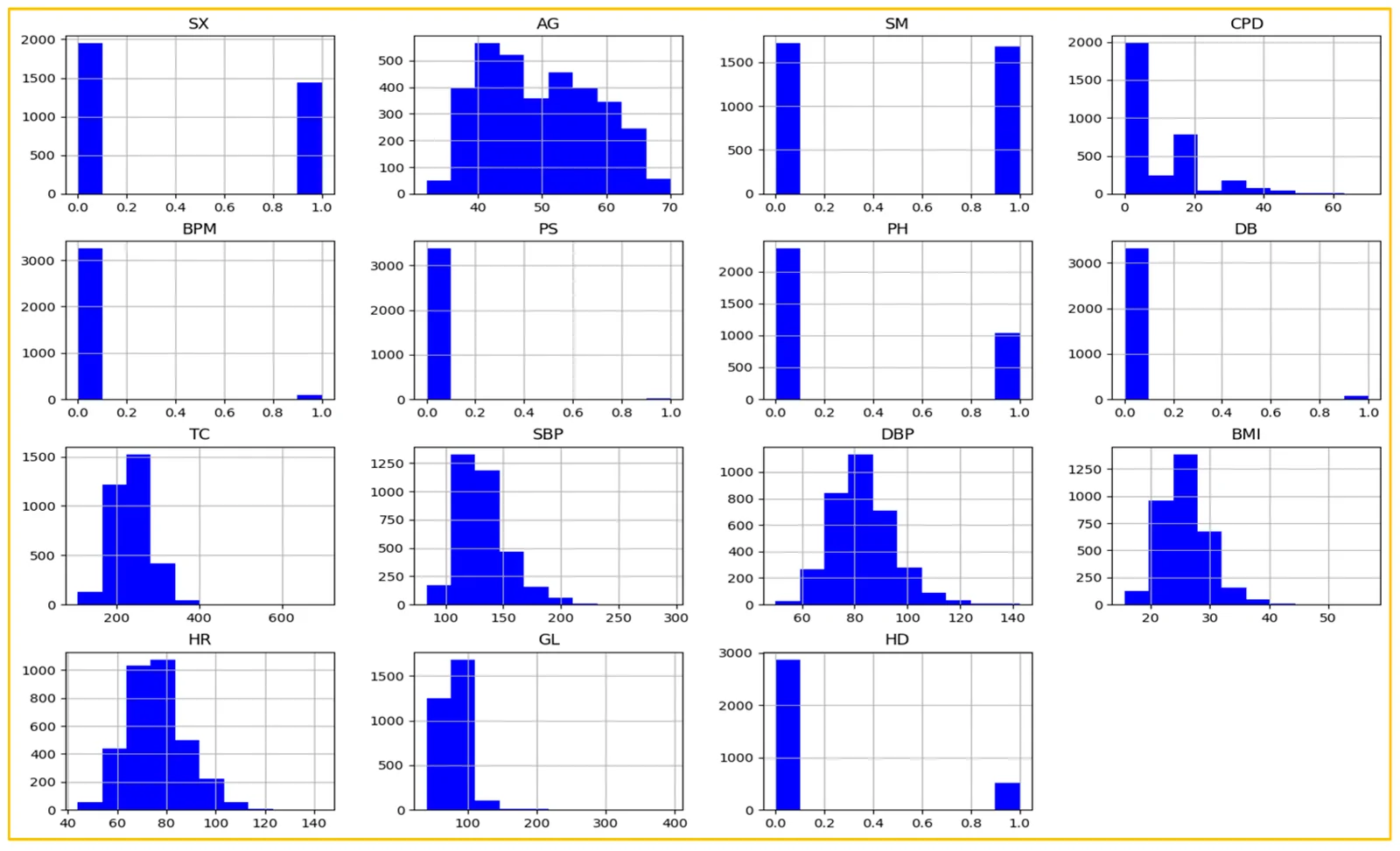
Distribution of variables in the heart disease dataset.

**Figure A2:**
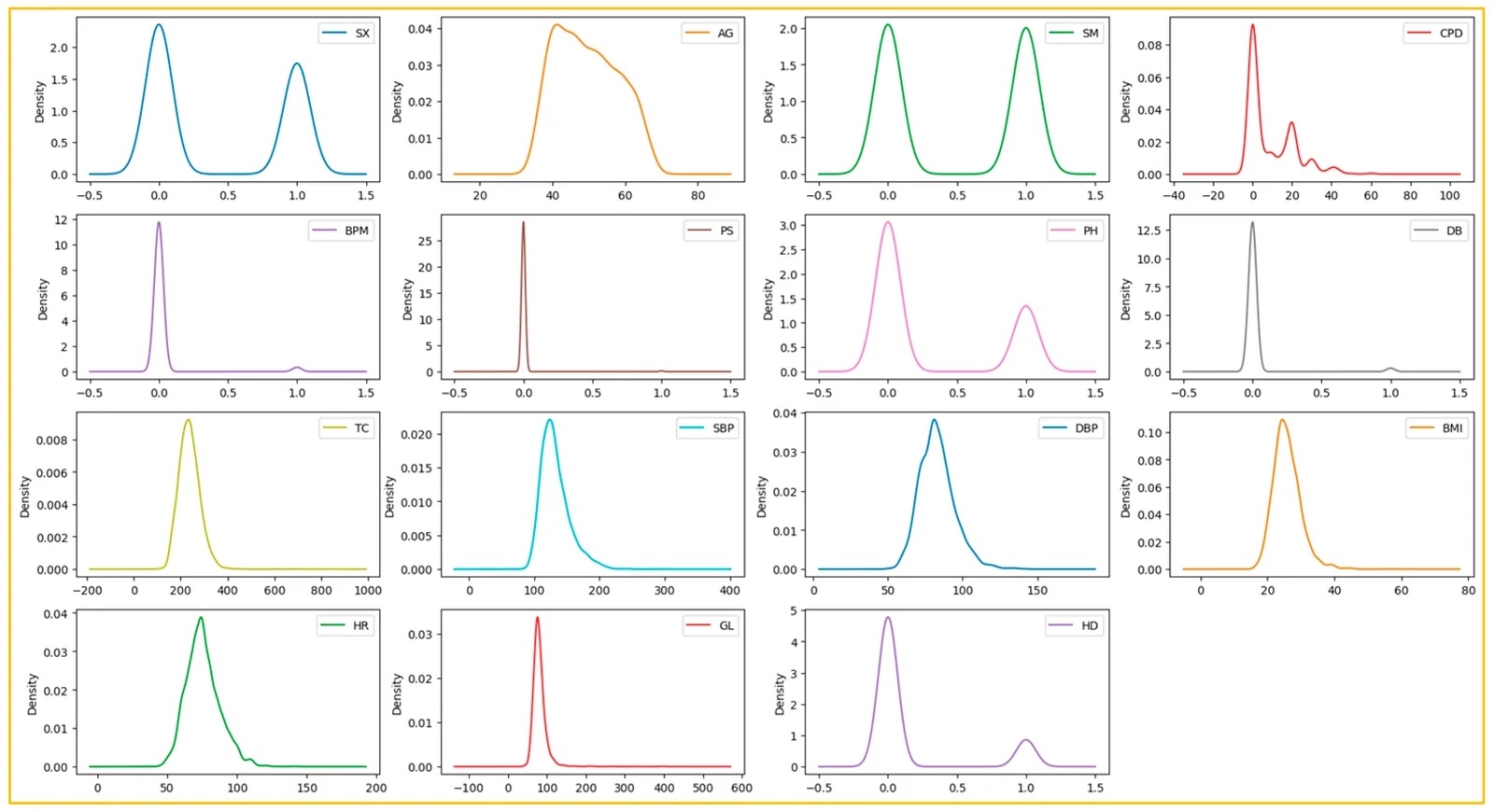
Kernel density estimates of numerical variables in the heart disease dataset.

**Figure A3:**
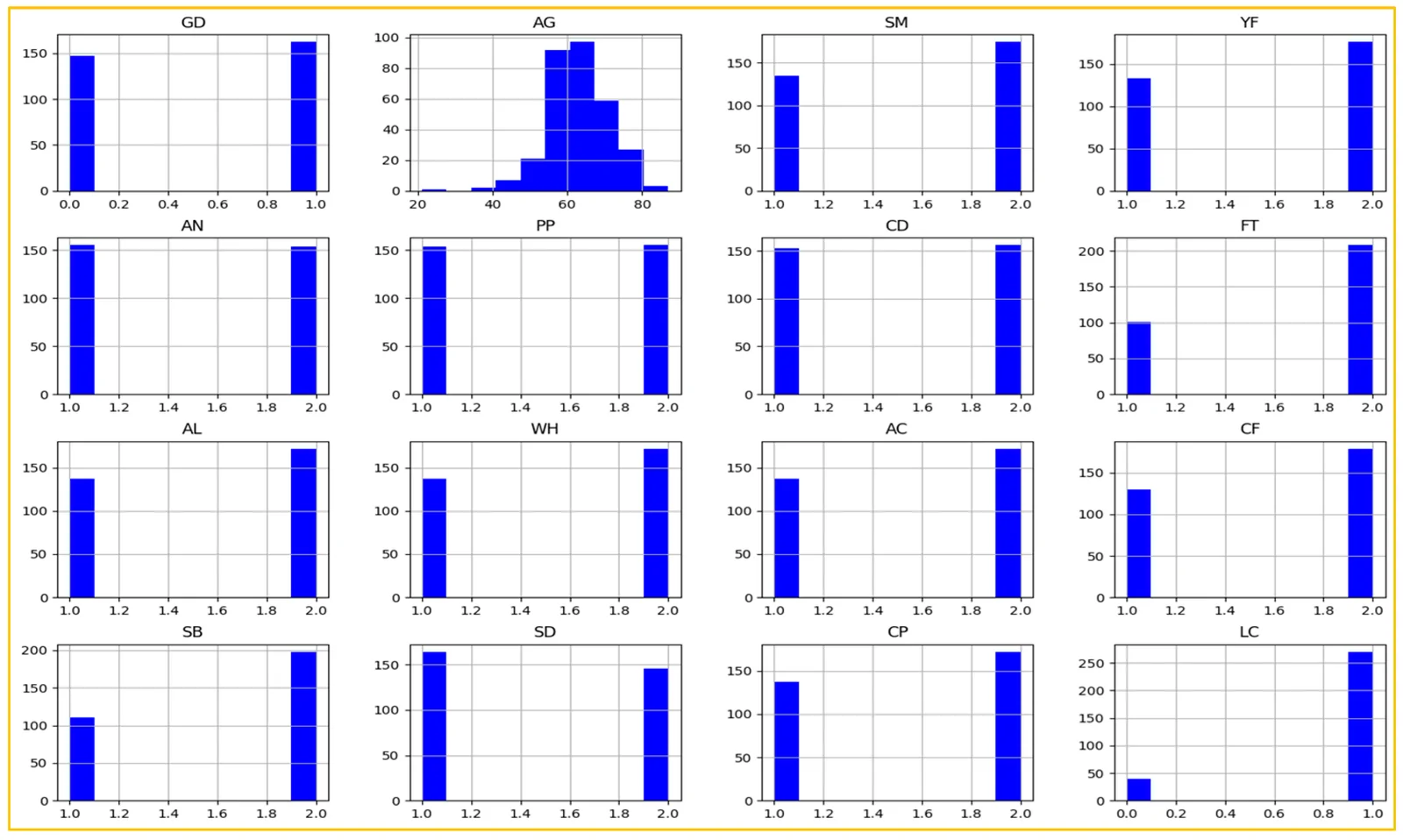
Distribution of variables in the lung cancer dataset.

**Figure A4:**
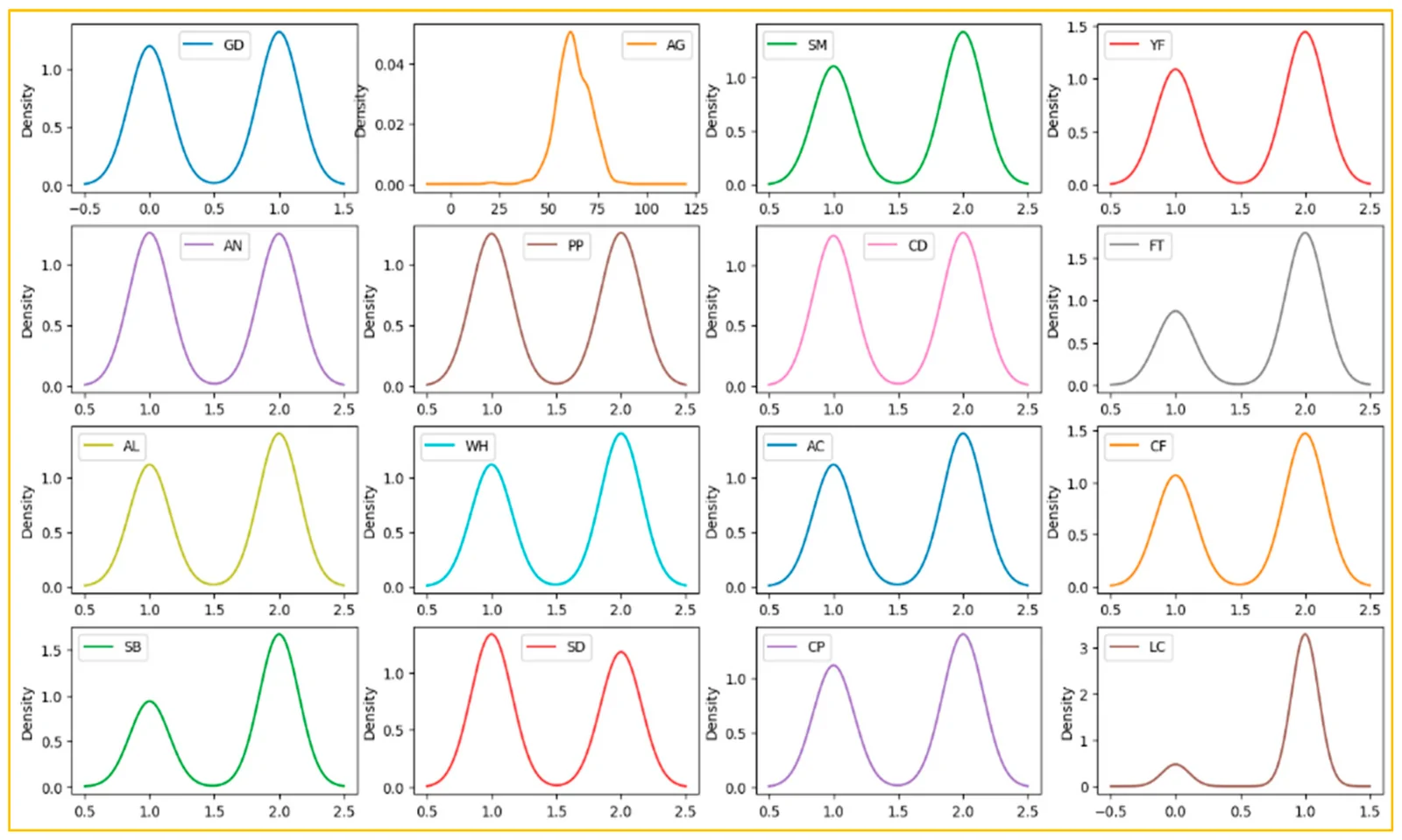
Distributional visualization of variables in the lung cancer dataset.

**Figure A5:**
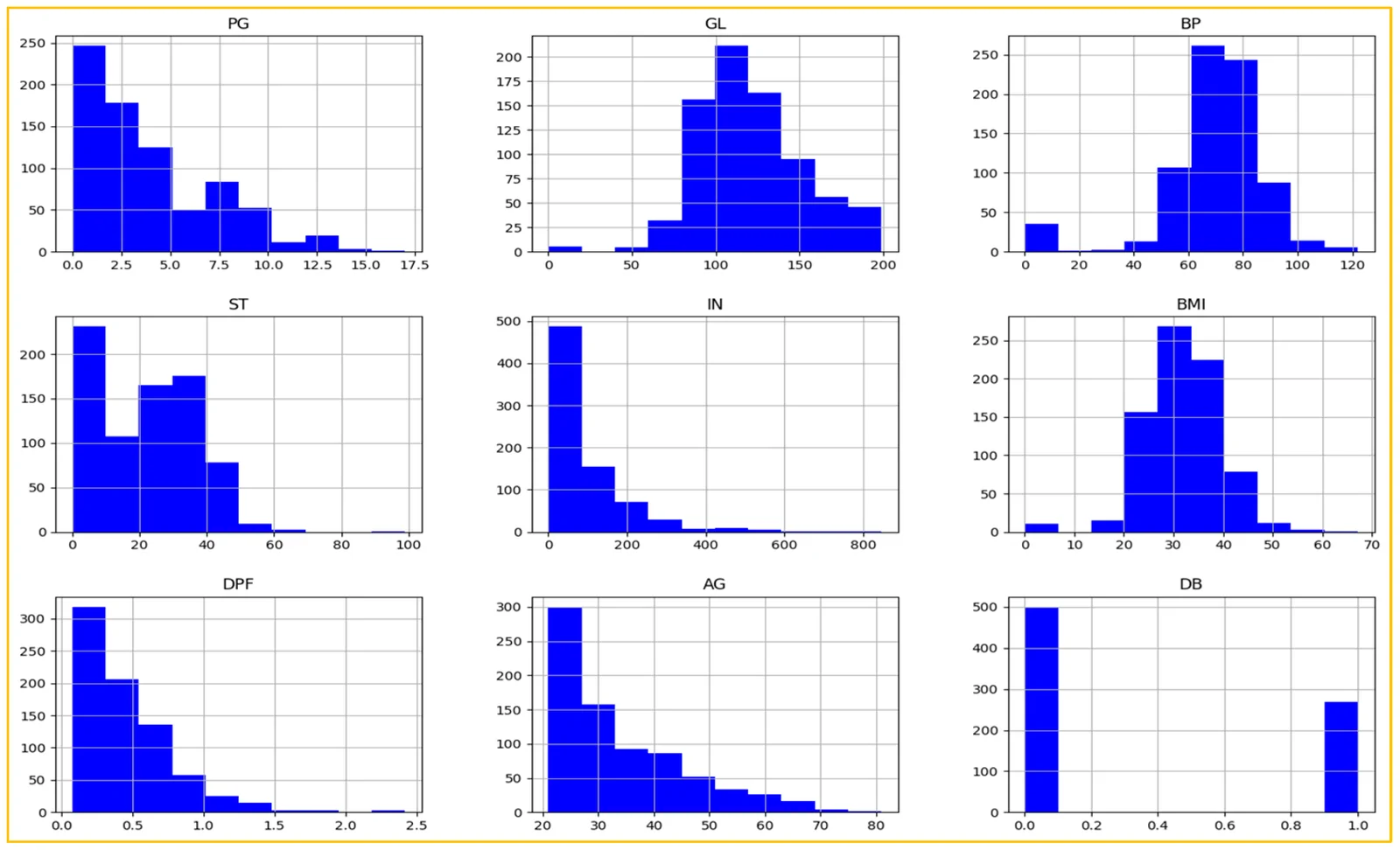
Distribution of variables in the diabetes dataset.

**Figure A6:**
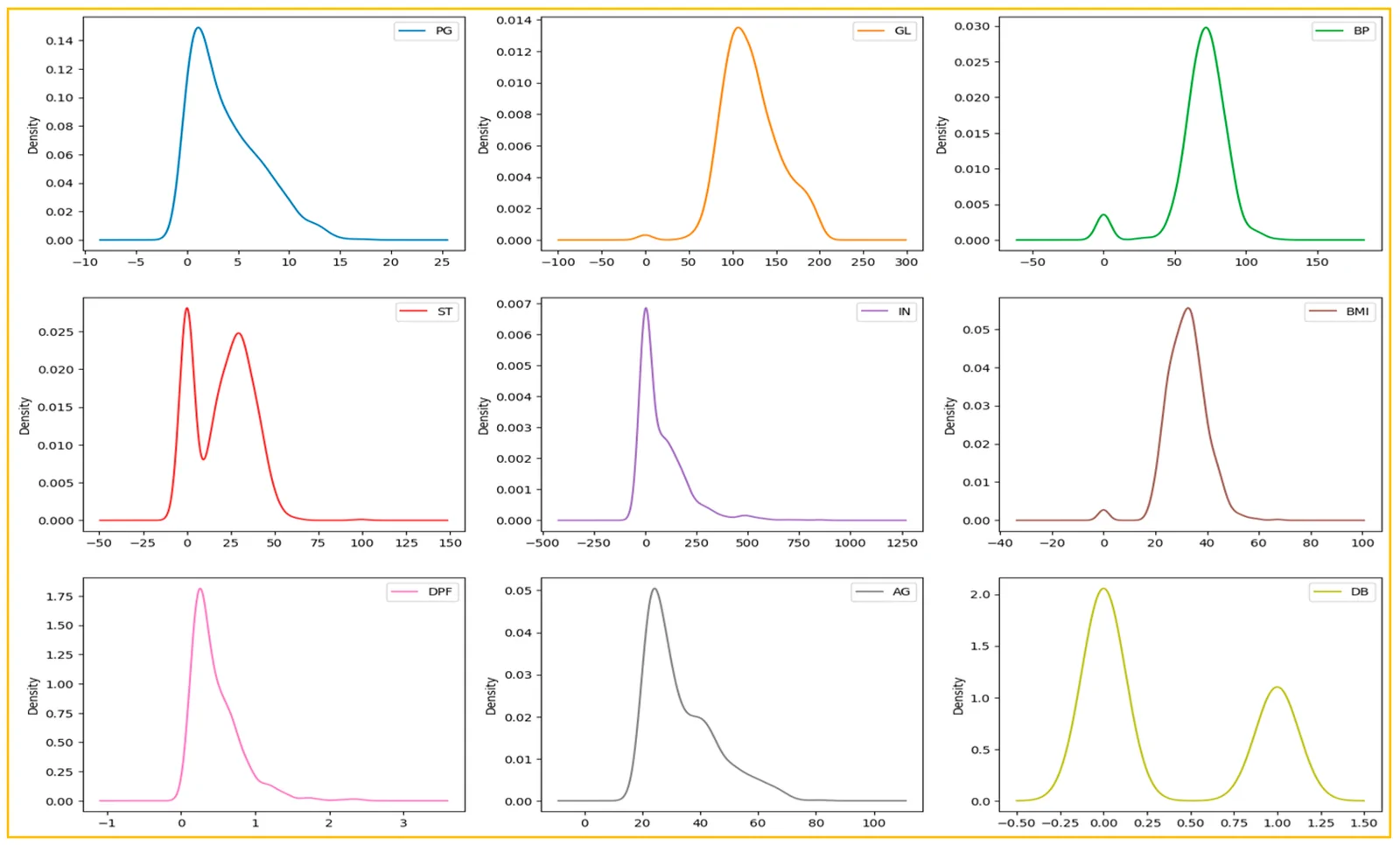
Kernel density estimates of numerical variables in the diabetes dataset.

EDA was performed to characterize the structure and distribution of the three disease-specific datasets before predictive modeling [48]. Numerical variables were examined using descriptive statistics, histograms, and kernel density estimates (KDEs), whereas categorical variables were examined using frequency-based visualizations. Correlation matrices were additionally used as descriptive tools to examine pairwise relationships among numerical or encoded variables. Potential extreme observations were inspected using distributional plots and the interquartile range (IQR) criterion. Because extreme physiological measurements may represent genuine observations rather than data errors, IQR-identified observations were not automatically removed. The class distribution of each binary outcome was also examined to quantify the extent of class imbalance. Supplementary distributional and class-frequency visualizations for the heart disease, lung cancer, and diabetes datasets are provided in Appendix A (Figure A1, Figure A2, Figure A3, Figure A4, Figure A5 and Figure A6), while the corresponding correlation matrices are retained in the main manuscript (Figure 3, Figure 4 and Figure 5).

Overall, the EDA demonstrated heterogeneous distributions across the three datasets, reflecting differences in their demographic, lifestyle, symptom-related, and physiological variables. Several continuous clinical variables exhibited skewness and extreme observations, while the binary outcomes showed varying degrees of class imbalance. Extreme observations were not removed solely on distributional grounds, as they could represent genuine physiological variation. The exploratory findings therefore informed a conservative preprocessing strategy in which missing-value handling, scaling, and class balancing were implemented within the training pipeline without altering the untouched held-out test distribution.

### 5.2. Data Preprocessing

**Figure 6:**
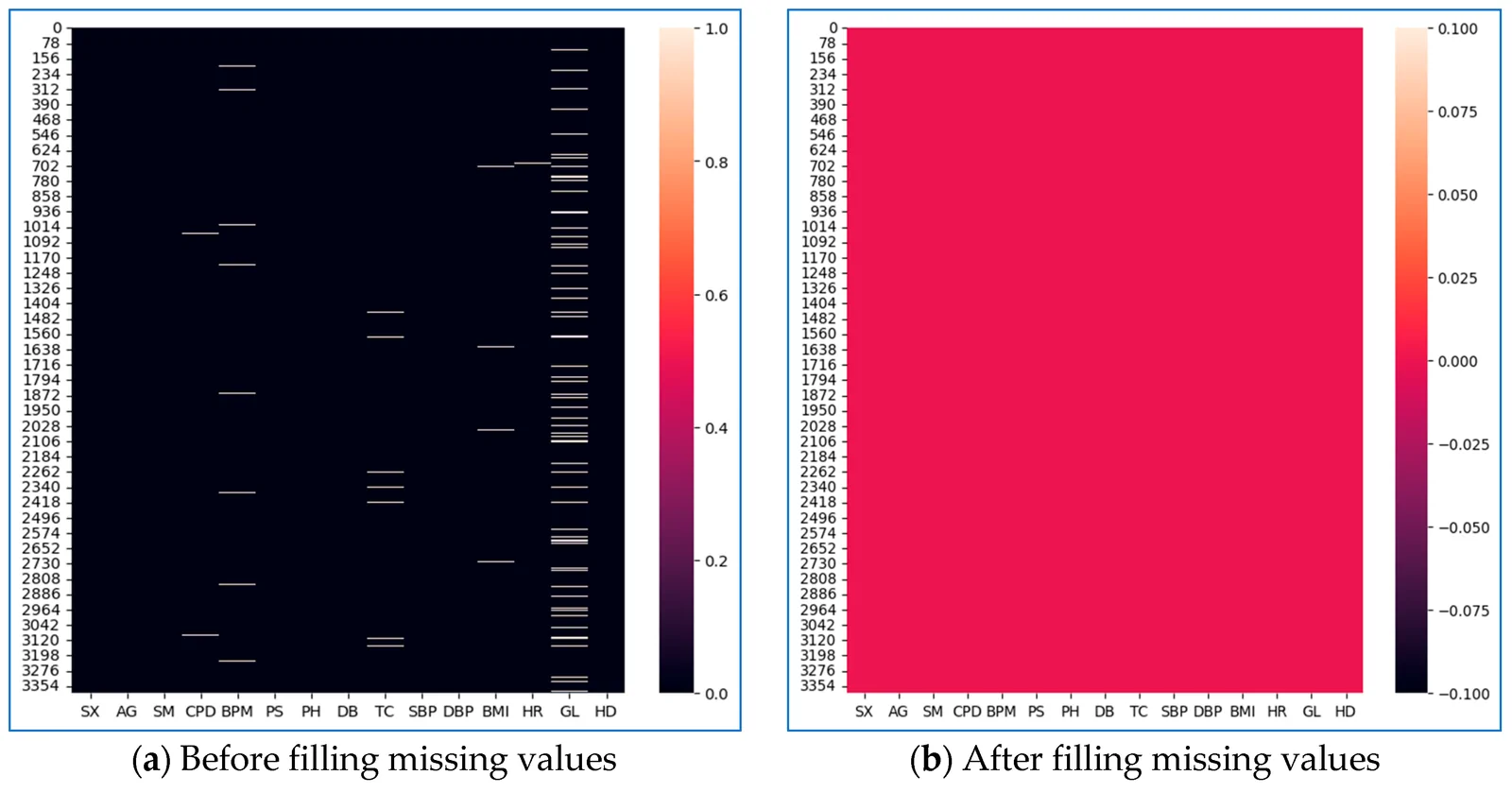
Illustration of missing-value handling in the heart disease dataset using median imputation.

Data preprocessing was performed within the model development pipeline after the initial stratified 80:20 train–test split. No fitted preprocessing operation was estimated using the held-out test data. For the heart disease and lung cancer datasets, missing numerical values were handled using median imputation, with the median values estimated independently from each training fold during cross-validation as shown in Figure 6. The imputed variables were subsequently transformed using MinMaxScaler, whose scaling parameters were likewise estimated exclusively from the corresponding training fold.

The diabetes dataset required an additional data-quality transformation. Zero values in glucose (GL), blood pressure (BP), skin thickness (ST), insulin (IN), and BMI were first converted to missing values because such zeros were considered non-physiological measurements in these variables. Zero values for pregnancies (PG) were retained because they represent a valid observation. Following this transformation, missing values were median-imputed and the predictors were scaled using MinMaxScaler. These operations were implemented sequentially within the training pipeline before random oversampling and model fitting.

**Figure 7:**
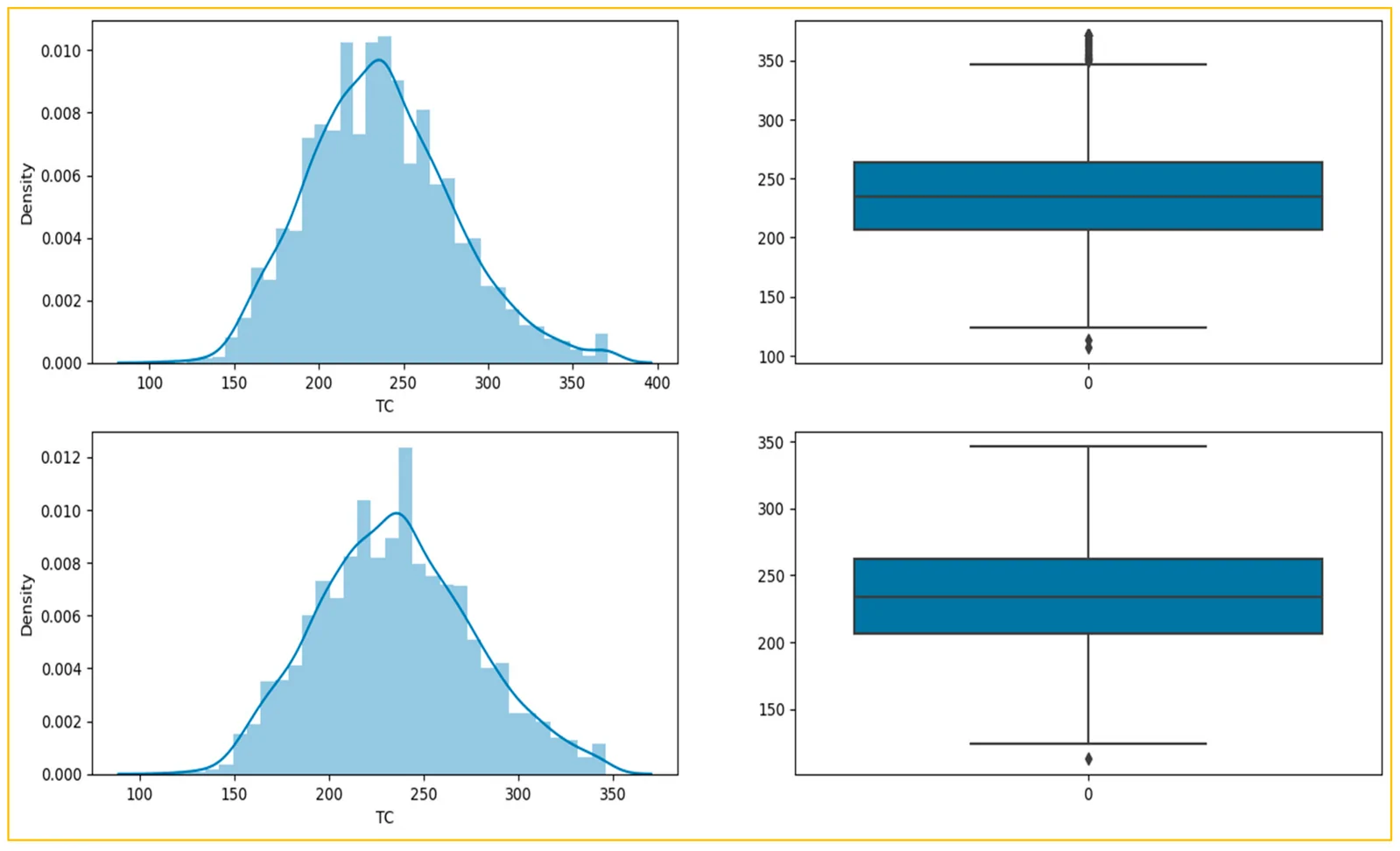
IQR-based outlier analysis of the Total Cholesterol attribute in the heart disease dataset.

**Figure 8:**
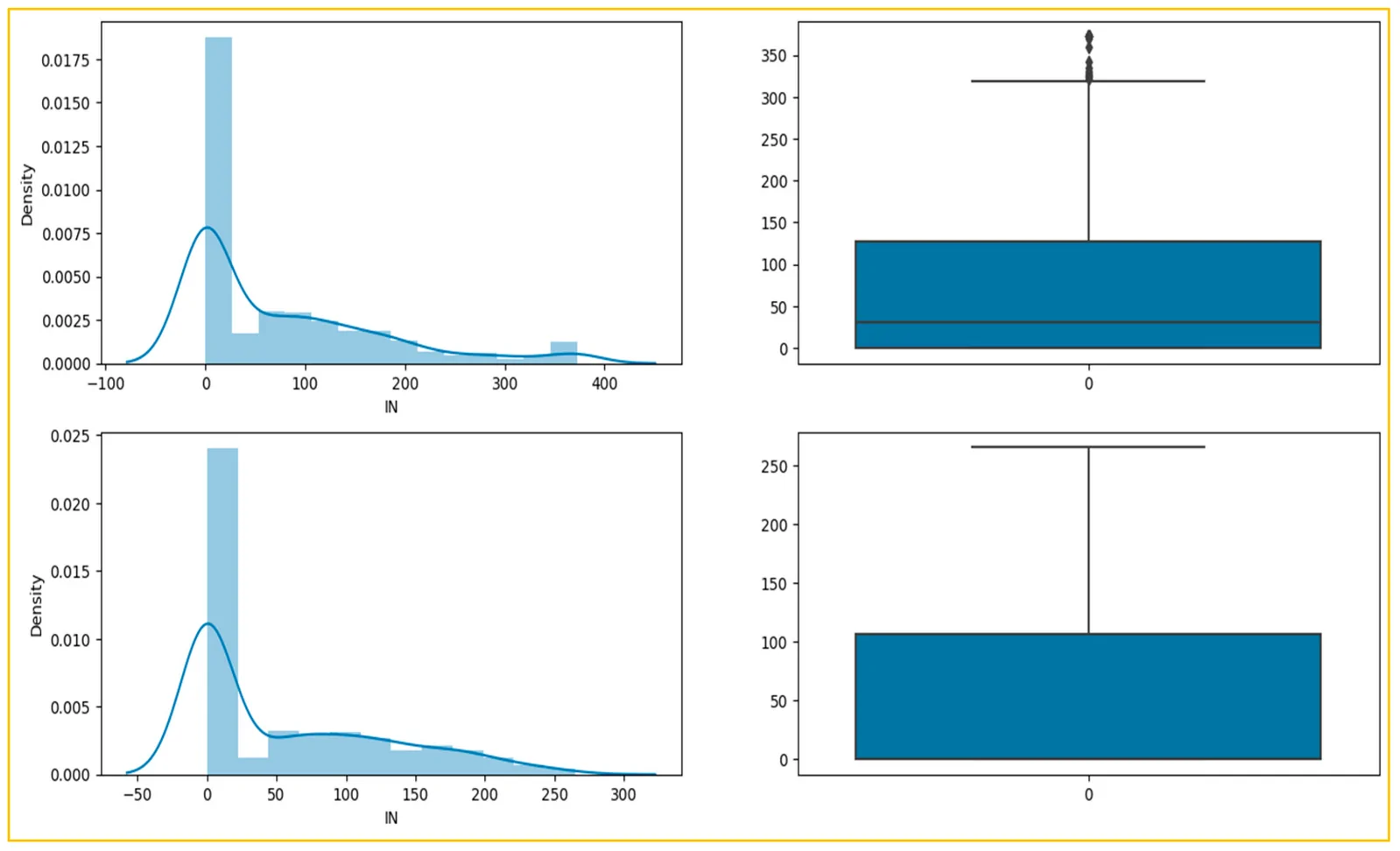
IQR-based outlier analysis of the Insulin attribute in the diabetes dataset.

Potential outliers were examined using the IQR criterion as part of the exploratory assessment, as shown in Figure 7 and Figure 8. However, observations were not automatically removed or replaced solely because they fell outside the IQR limits. Extreme values may represent genuine clinical or physiological variability, and indiscriminate removal could eliminate informative high-risk observations. Consequently, the IQR analysis was used to characterize potentially extreme measurements rather than as an automatic data-cleaning rule.

### 5.3. Class Balance

The three disease-specific datasets exhibited unequal binary class distributions, with the most pronounced imbalance observed in the lung cancer dataset. To reduce the influence of class imbalance during model training, random oversampling of the minority class was performed using RandomOverSampler [49]. Importantly, oversampling was applied only to the training portion of each cross-validation fold after preprocessing and was never applied before the initial train–test partition. The corresponding validation fold and the final held-out test set retained their original class distributions. This design prevents duplicate minority-class observations generated during oversampling from appearing simultaneously in both the model training and evaluation subsets.

**Figure 9:**
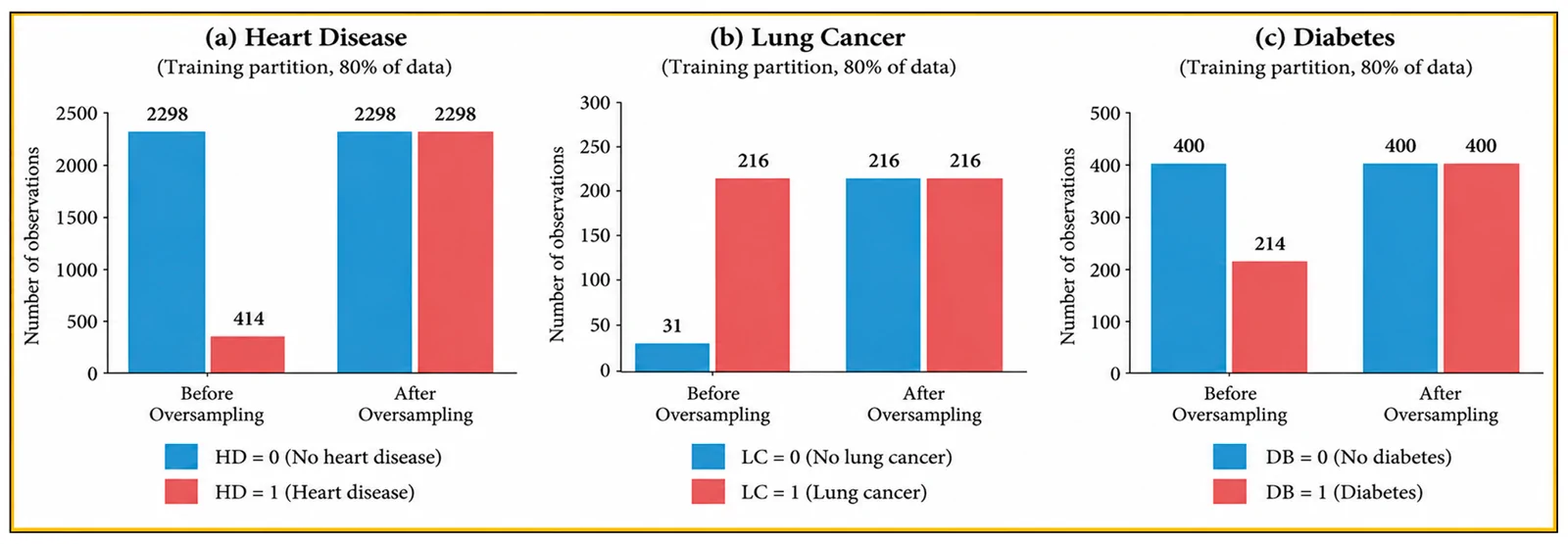
Class distributions before and after random oversampling for the heart disease, lung cancer, and diabetes training partitions.

Random oversampling was used instead of synthetic interpolation. This choice preserved observed minority-class feature combinations while avoiding the generation of artificial intermediate observations across variables that include binary, categorical, ordinal-coded, and continuous clinical characteristics, as shown in Figure 9. The oversampling procedure was therefore used exclusively as a training-stage class-balancing operation and did not modify the prevalence of either class in the held-out test sets.

## 6. Experimental Results and Performance Evaluation

Machine learning and ensemble learning techniques yielded better results for decision-making across multiple domains and sectors, especially healthcare [50,51,52]. This section presents the results obtained under leakage-free experimental protocol. Performance was evaluated independently for the heart disease, lung cancer, and diabetes prediction tasks using the untouched 20% test partitions. For each model, the decision threshold was determined exclusively from calibrated out-of-fold predictions generated from the corresponding training set and was locked before test-set evaluation. Consequently, the results reported below reflect performance at the training-derived decision thresholds rather than at an arbitrary probability threshold of 0.50. Accuracy, balanced accuracy, precision, sensitivity, specificity, F1-score, ROC-AUC, PR-AUC, and Brier score were evaluated, along with 95% bootstrap confidence intervals. Repeated stratified cross-validation was additionally used as a supplementary analysis of model stability and comparative performance.

### 6.1. Held-Out Test Performance

The five boosting algorithms were first evaluated on the disease-specific held-out test sets using their locked training-derived thresholds. Because the datasets differed substantially in class distribution, model comparison was not based on accuracy alone. Balanced accuracy was emphasized for assessing the trade-off between sensitivity and specificity, while ROC-AUC and PR-AUC quantified threshold-independent discrimination. The Brier score was additionally used to assess probabilistic prediction quality, with lower values indicating better performance.

#### 6.1.1. Heart Disease

Table 4 summarizes the performance of the five boosting algorithms on the untouched heart disease test set using the locked thresholds derived from training-only out-of-fold predictions. The results demonstrate a substantial trade-off between overall accuracy and minority-class detection. AdaBoost achieved the highest balanced accuracy of 68.07%, with a sensitivity of 67.96% and specificity of 68.17%. It also obtained the highest F1-score (39.33%) and ROC-AUC (71.71%) among the evaluated models. Gradient Boosting showed comparable balanced accuracy (67.62%) and F1-score (39.08%), while CatBoost achieved a balanced accuracy of 66.91%. LightGBM produced the highest overall accuracy (70.35%) and specificity (72.00%), but its sensitivity was lower at 61.17%. These findings illustrate why overall accuracy alone is insufficient for this imbalanced task.

**Figure 10:**
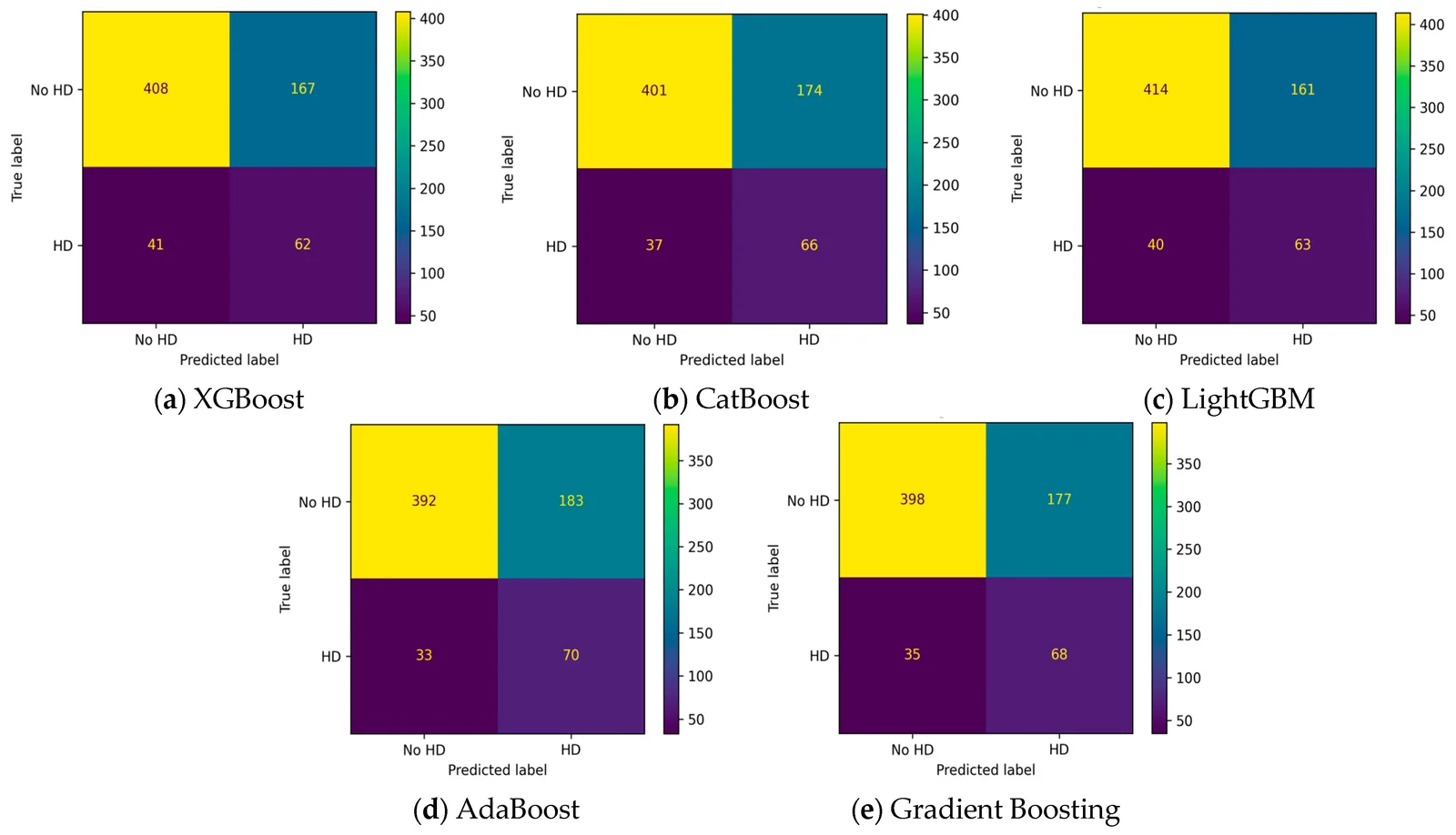
Confusion matrices of the five boosting models on the heart disease test set.

The confusion matrices further demonstrate this trade-off as shown in Figure 10. At its locked threshold of 0.1617, AdaBoost correctly identified 70 of 103 heart-disease-positive observations and 392 of 575 negative observations. Gradient Boosting correctly identified 68 positive cases, whereas CatBoost, LightGBM, and XGBoost identified 66, 63, and 62, respectively. No single model simultaneously maximized sensitivity and specificity, supporting the use of balanced accuracy and multiple complementary metrics for interpretation.

**Figure 11:**
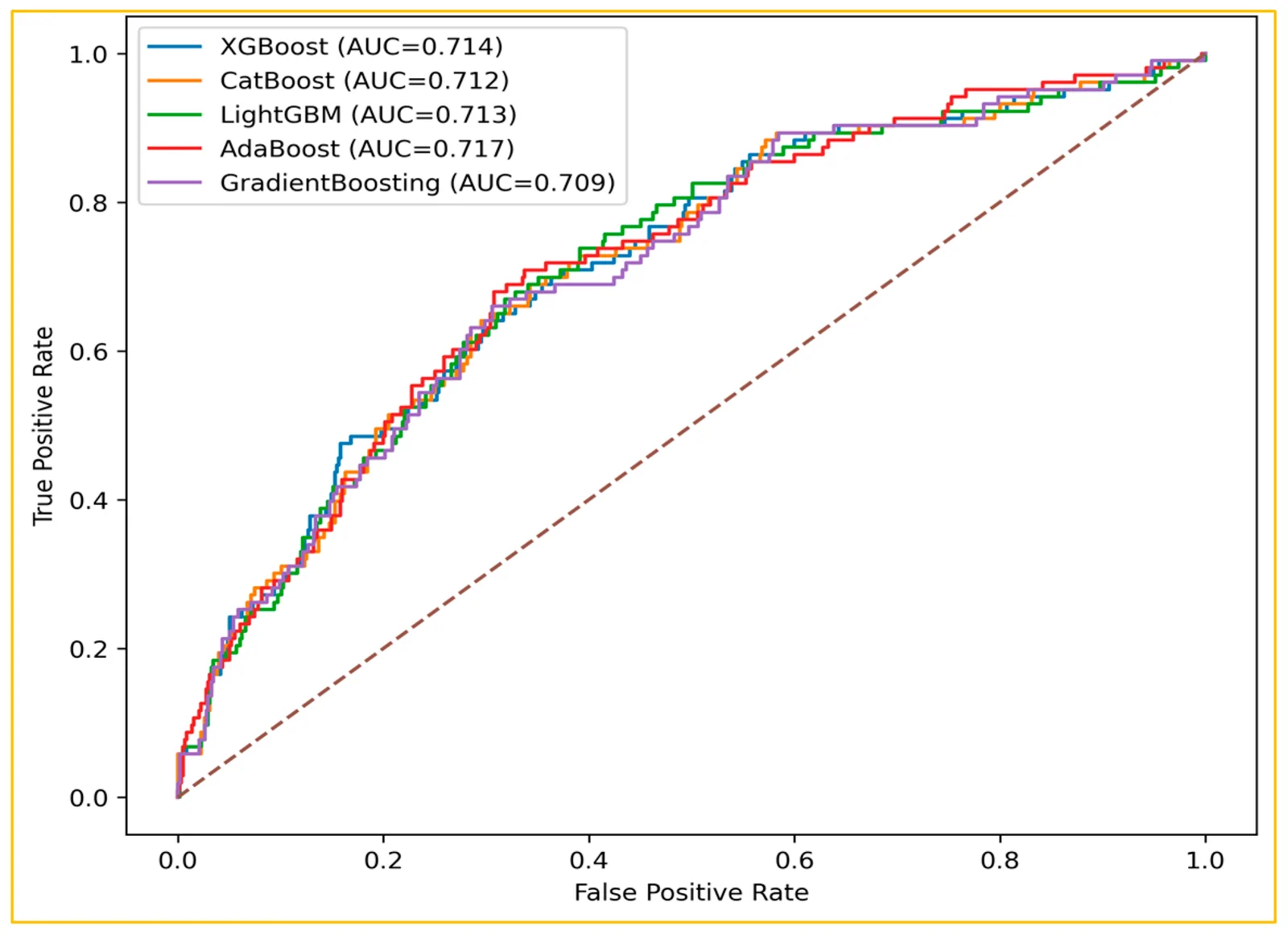
ROC curve of the five boosting models for heart disease.

**Figure 12:**
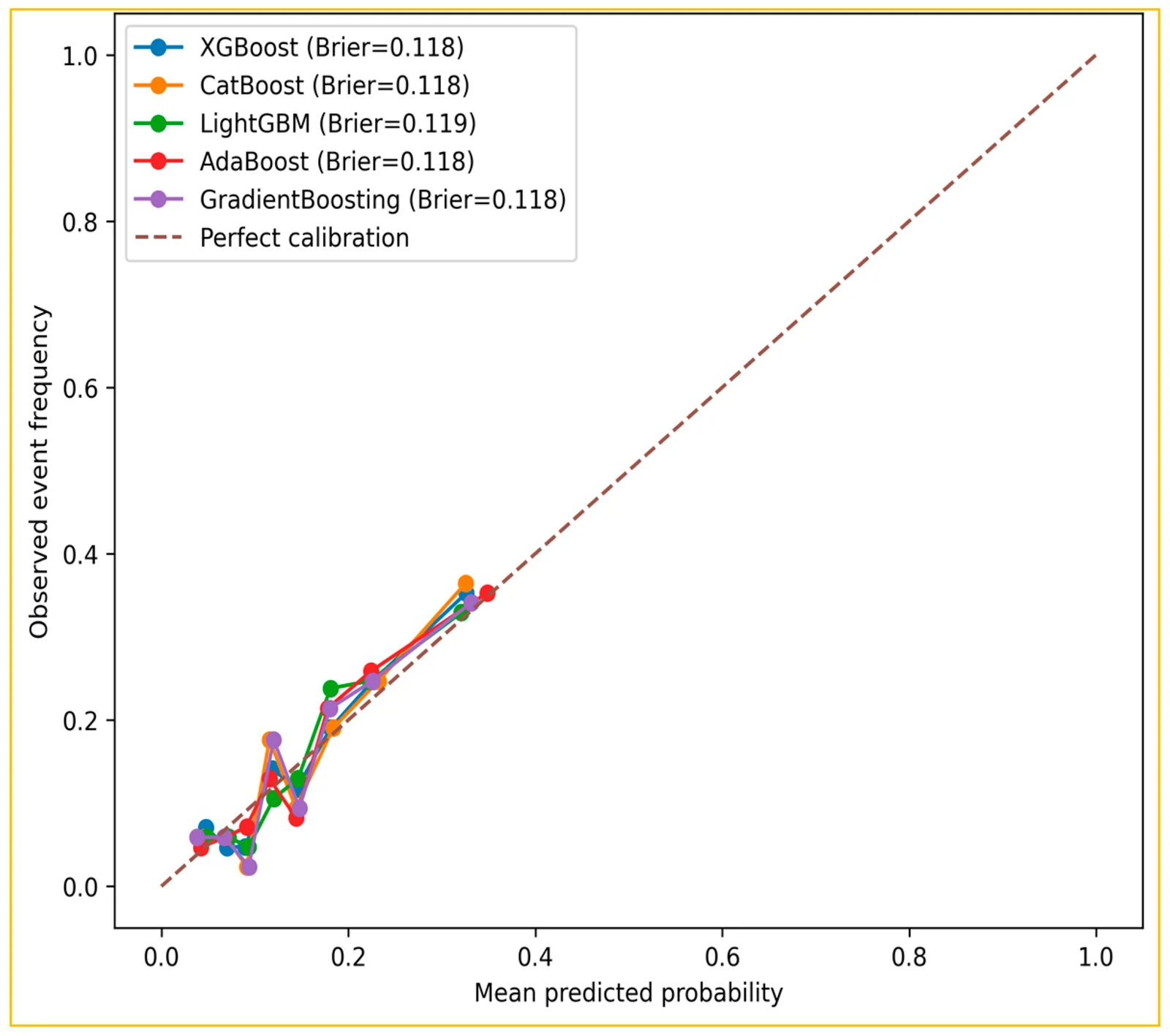
Probability–calibration curve of the five boosting models for heart disease.

ROC-AUC values were relatively close across models, ranging from 70.92% to 71.71%, indicating broadly similar discrimination as presented in Figure 11. PR-AUC ranged from 32.07% to 33.88%, reflecting the relatively low prevalence of the positive class. Brier scores were also similar (approximately 0.118), with AdaBoost obtaining the lowest value (0.1177) as presented in Figure 12. The bootstrap intervals indicate that differences among individual models on this single held-out test set should be interpreted cautiously.

#### 6.1.2. Lung Cancer

Table 5 presents the held-out lung cancer results. CatBoost achieved the strongest overall performance at its training-derived threshold, with a balanced accuracy of 92.59%, sensitivity of 85.19%, specificity of 100%, F1-score of 92.00%, and ROC-AUC of 96.30%. LightGBM followed closely with a balanced accuracy of 91.67% and ROC-AUC of 96.06%, while XGBoost achieved a balanced accuracy of 89.81%.

**Figure 13:**
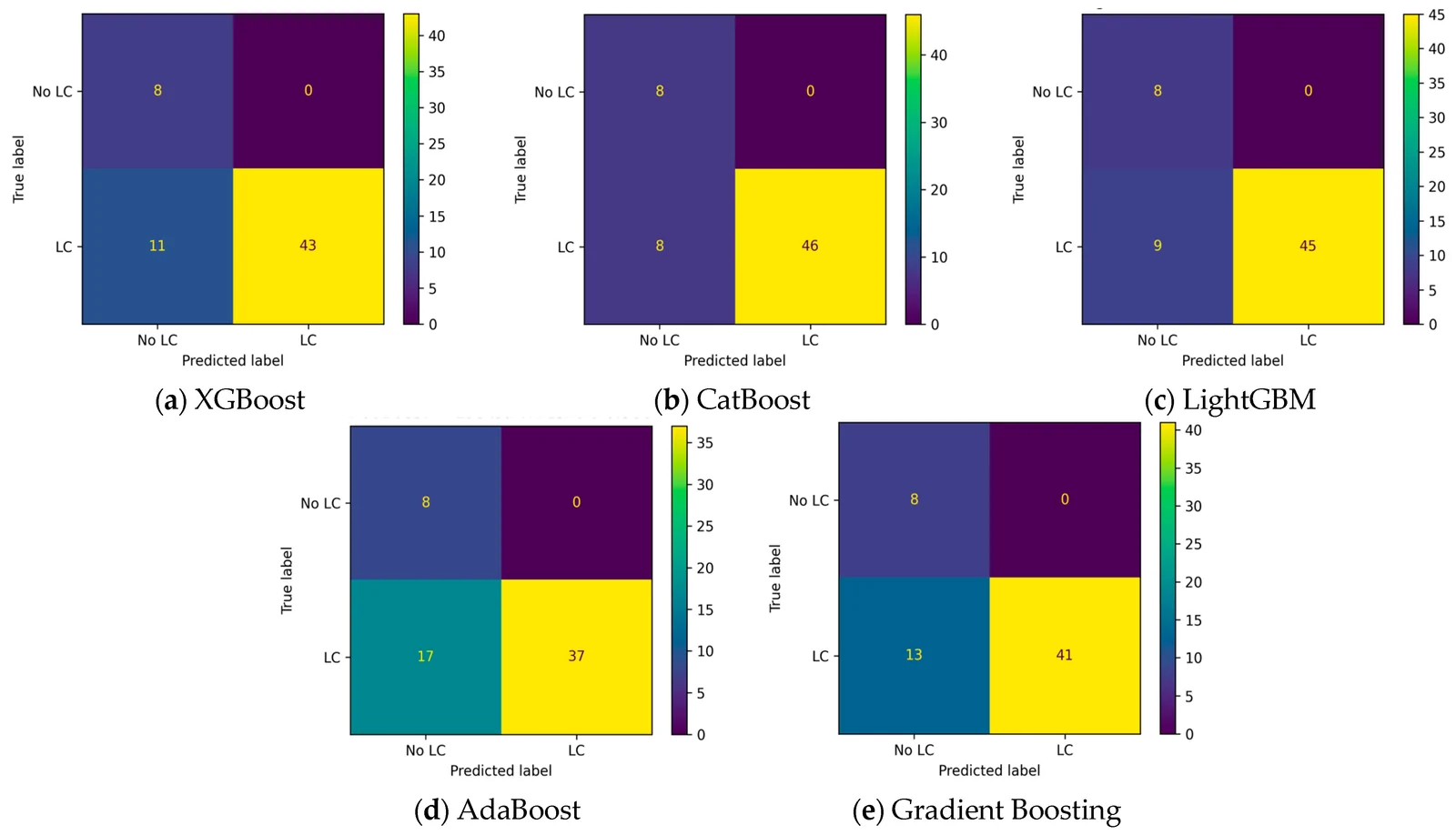
Confusion matrices of the five boosting models on the lung cancer test set.

All five models achieved 100% specificity at their optimized thresholds, meaning that all eight lung-cancer-negative observations in the held-out test set were correctly classified. However, this result must be interpreted cautiously because specificity was estimated from only eight negative observations as depicted in Figure 13. Thus, the apparent perfect specificity should not be interpreted as evidence that the models would maintain 100% specificity in a larger or clinically representative population. The principal differences among models resulted from their sensitivity to lung-cancer-positive cases: CatBoost correctly identified 46 of 54 positive observations, LightGBM 45, XGBoost 43, Gradient Boosting 41, and AdaBoost 37.

**Figure 14:**
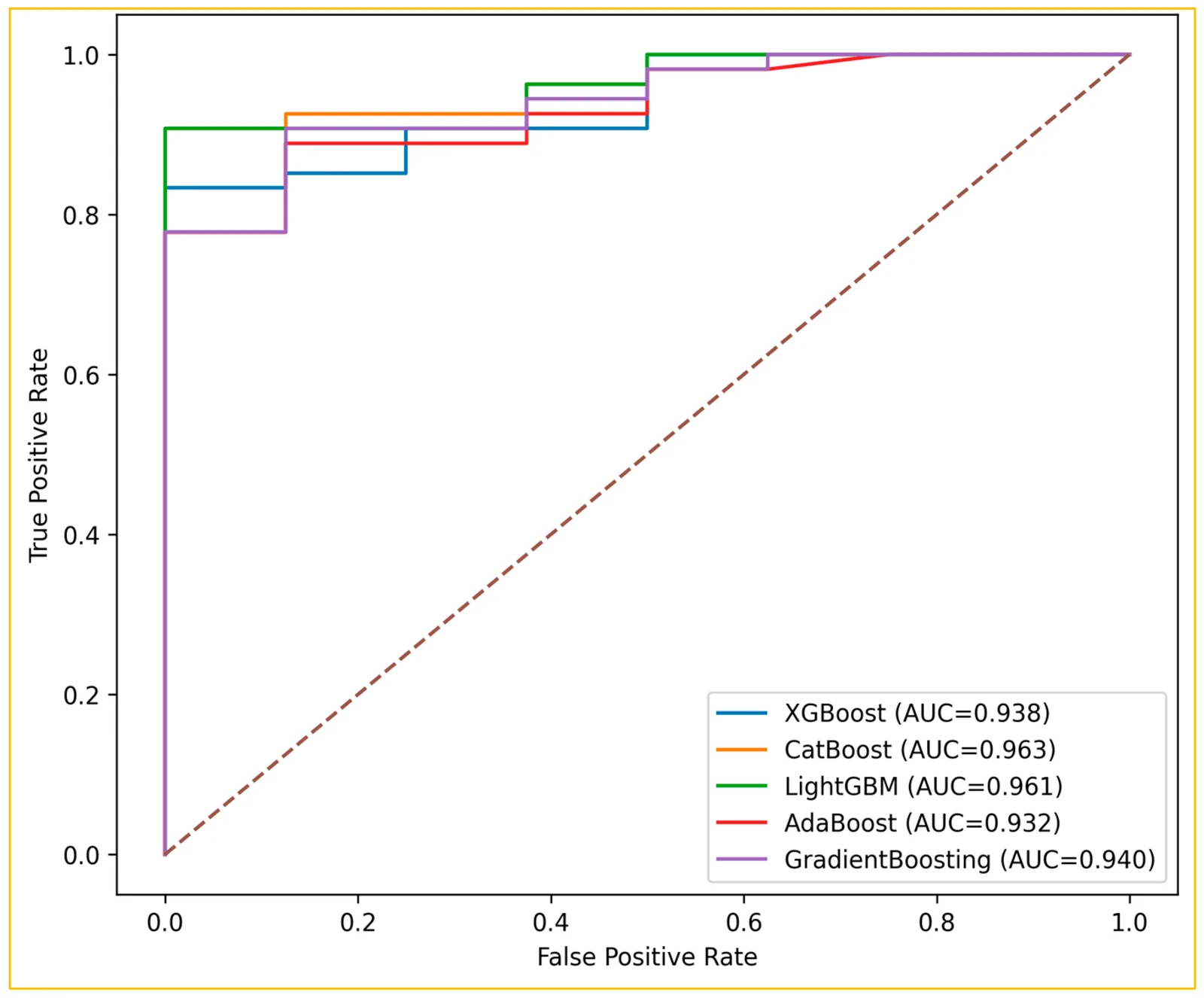
ROC curve of the five boosting models for lung cancer.

**Figure 15:**
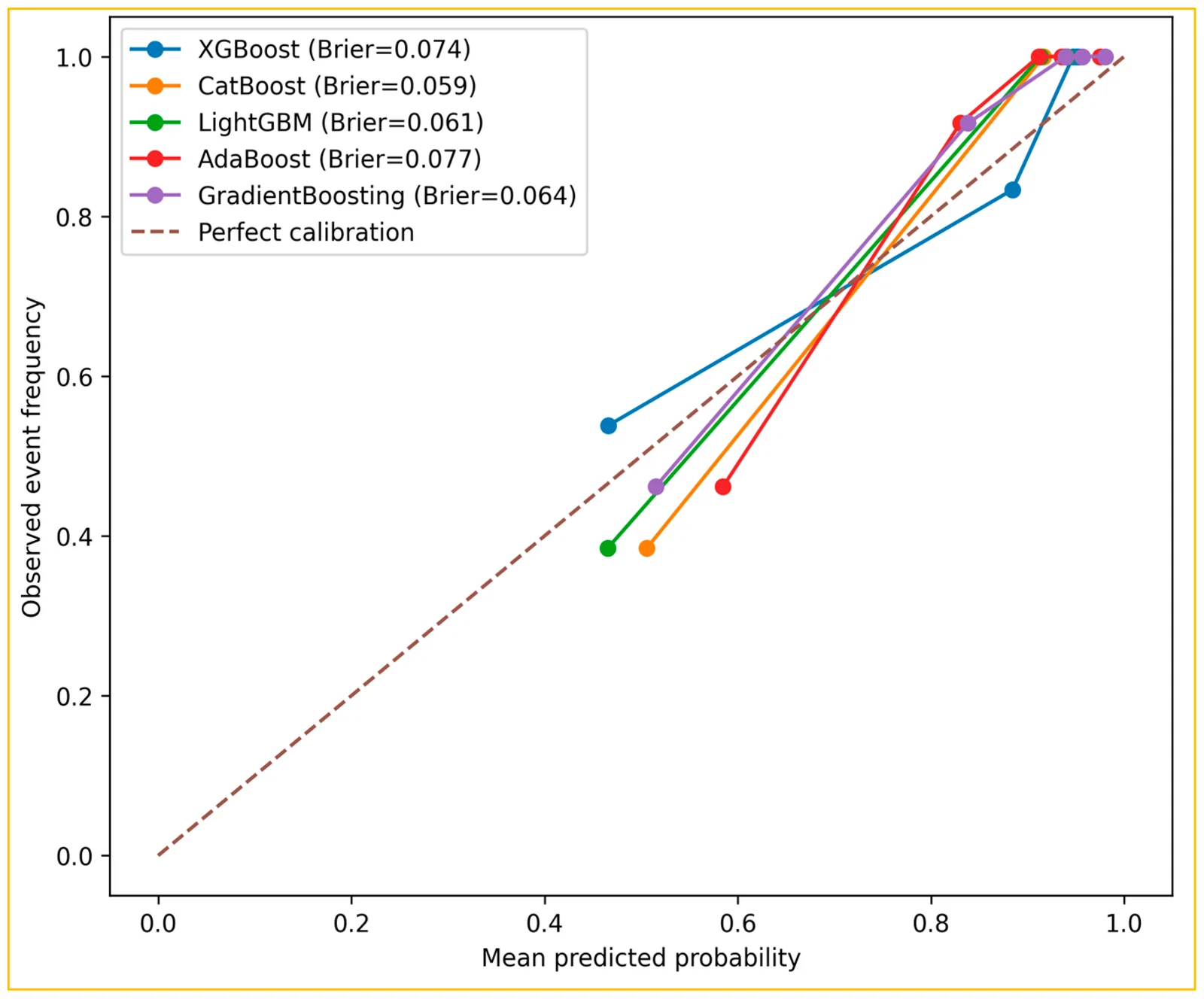
Probability–calibration curve of the five boosting models for lung cancer.

As depicted in Figure 14 and Figure 15, CatBoost also produced the lowest Brier score (0.0593) and highest PR-AUC (99.47%), although the extremely high PR-AUC partly reflects the high prevalence of the positive class in this benchmark dataset. The bootstrap confidence intervals remained relatively wide for several measures because the held-out test set comprised only 62 observations, further emphasizing the need for cautious interpretation.

#### 6.1.3. Diabetes

**Figure 16:**
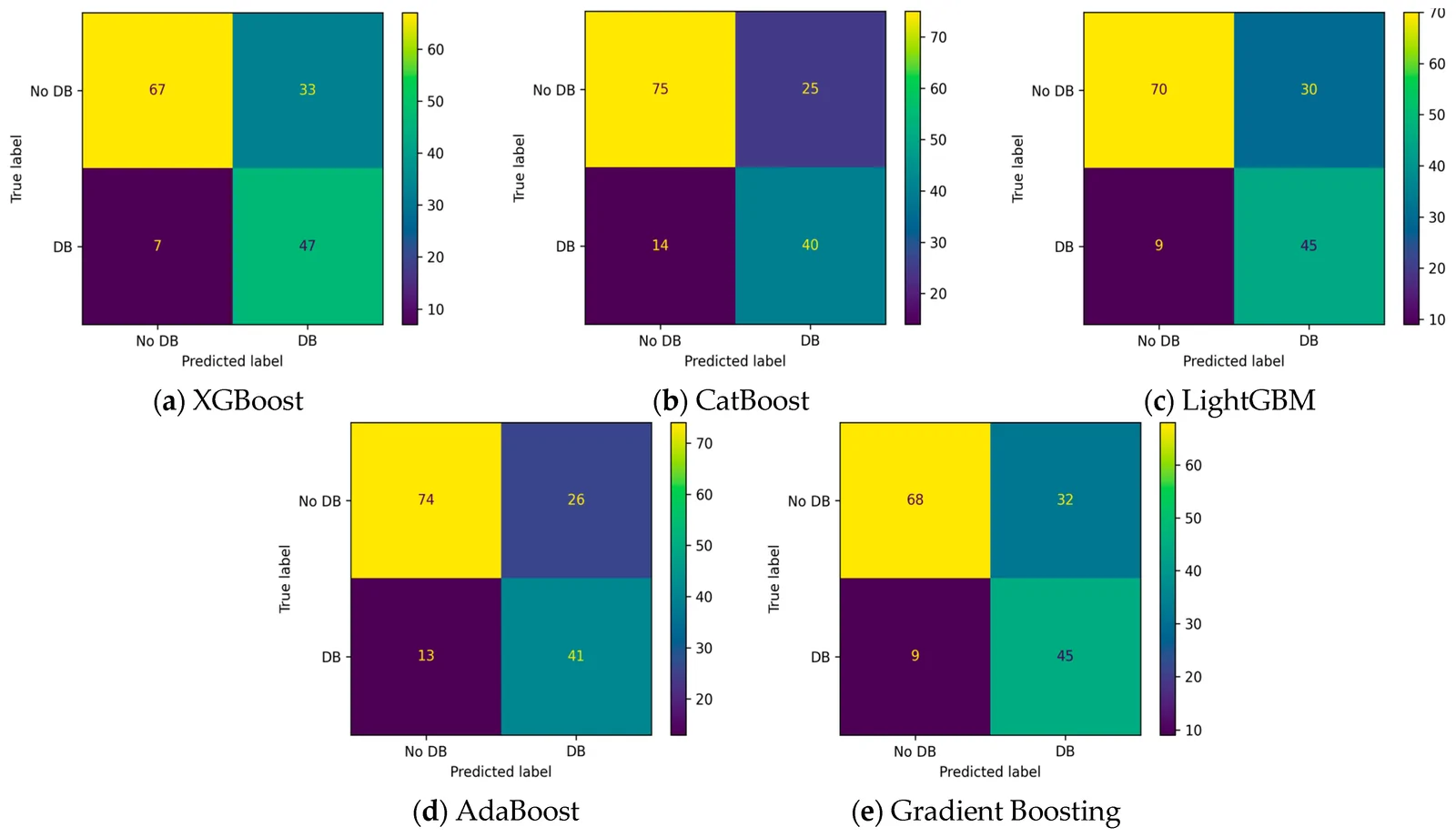
Confusion matrices of the five boosting models on the diabetes test set.

**Figure 17:**
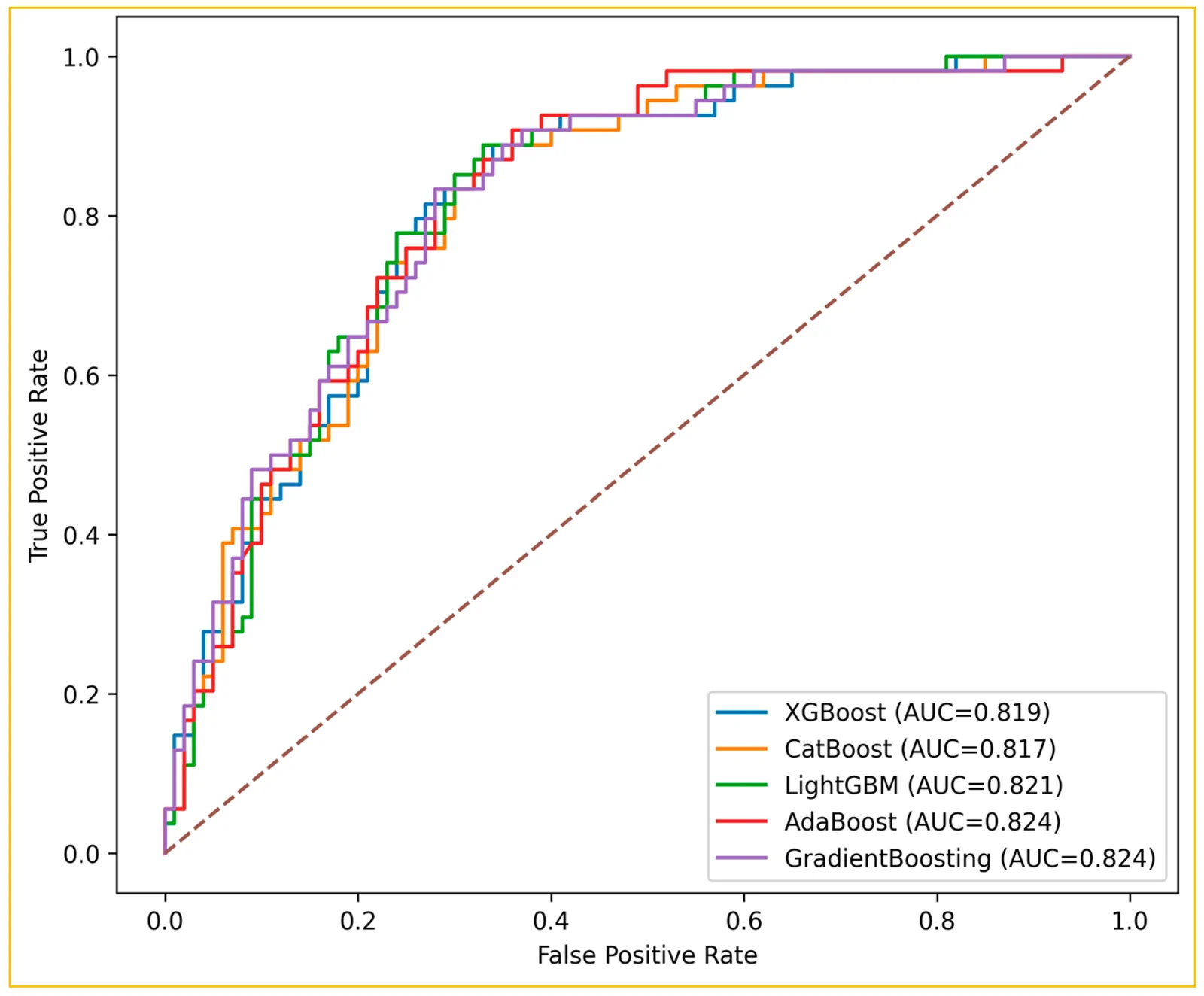
ROC curve of the five boosting models for diabetes.

**Figure 18:**
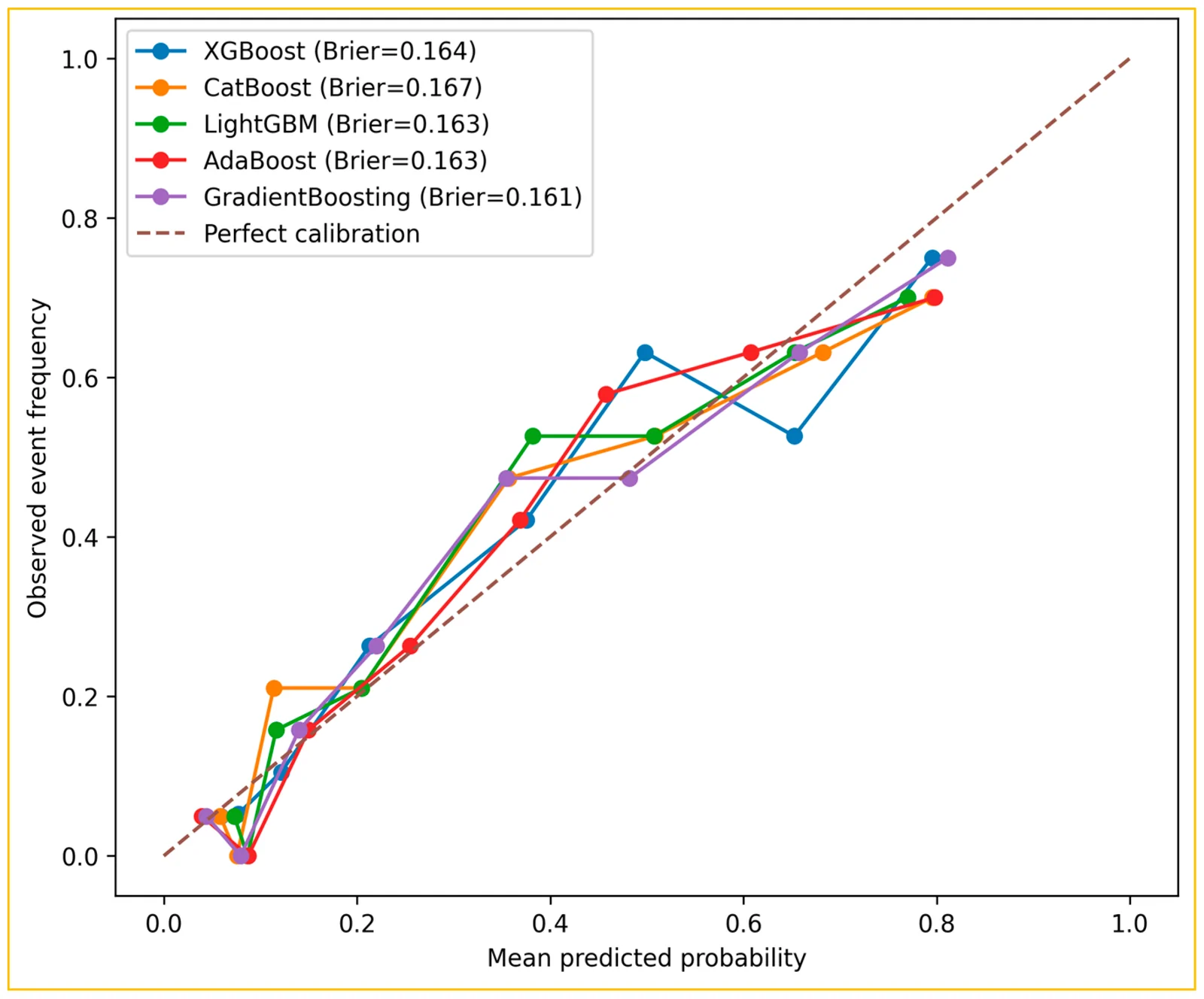
Probability–calibration curve of the five boosting models for diabetes.

The held-out diabetes results are summarized in Table 6. XGBoost achieved the highest balanced accuracy of 77.02%, primarily because of its high sensitivity (87.04%), although its specificity was comparatively lower (67.00%). LightGBM obtained a similar balanced accuracy of 76.67%, with sensitivity of 83.33% and specificity of 70.00%. In terms of threshold-dependent classification, XGBoost correctly identified 47 of 54 diabetic observations, corresponding to only seven false negatives, but generated 33 false positives among the 100 non-diabetic observations, as shown in Figure 16. LightGBM and Gradient Boosting each identified 45 diabetic cases. CatBoost showed the highest specificity (75.00%) among the optimized-threshold models, but its sensitivity was lower (74.07%). It can be observed from Figure 17 and Figure 18 that Gradient Boosting achieved a balanced accuracy of 75.67% but produced the highest ROC-AUC (82.43%) and PR-AUC (69.70%) as well as the lowest Brier score (0.1614). These results again demonstrate that model ranking depends on the evaluation endpoint rather than on a single measure of performance.

### 6.2. Cross-Validation and Statistical Comparison

To complement the single held-out evaluations, model stability and relative performance were further examined using five-fold stratified cross-validation repeated five times for each disease dataset. To avoid treating correlated folds as independent observations, the five folds within each repetition were averaged. This resulted in 15 matched dataset–repeat blocks across the three datasets. Balanced accuracy was designated as the primary statistical endpoint, whereas ROC-AUC and F1-score were analyzed as secondary endpoints.

#### 6.2.1. Friedman Omnibus Analysis

The Friedman test (https://tec.citius.usc.es/stac/index.html accessd on 22 May 2026) identified statistically significant differences among the five algorithms for all three endpoints as presented in Table 7. For balanced accuracy, the test yielded $\chi^{2}(4)=26.293,p<0.001$. Significant overall differences were also observed for ROC-AUC, $\chi^{2}(4)=21.173,p<0.001$, and F1-score, $\chi^{2}(4)=15.093,\;p=0.0045$. These results indicate that the algorithms did not exhibit equivalent relative performance across the matched dataset–repeat blocks.

#### 6.2.2. Post Hoc Pairwise Analysis and Model Ranking

Because each omnibus test was significant, all ten model pairs were compared using two-sided Wilcoxon signed-rank tests [53], followed by Holm correction [54] within each endpoint. As presented in Table 8 and Table 9, for balanced accuracy, statistically significant paired differences were observed between AdaBoost and LightGBM (Holm-adjusted *p* = 0.0342), Gradient Boosting and LightGBM (*p* = 0.00061), and Gradient Boosting and XGBoost (*p* = 0.00110). For ROC-AUC, Gradient Boosting showed significantly higher paired values than LightGBM (*p* = 0.00061) and XGBoost (*p* = 0.00110), while XGBoost showed significantly higher paired values than LightGBM (*p* = 0.0122). For F1-score, CatBoost showed significantly higher paired values than AdaBoost (*p* = 0.0201), LightGBM (*p* = 0.0269), and XGBoost (*p* = 0.0201), while Gradient Boosting showed significantly higher paired values than AdaBoost (*p* = 0.0376). Rank-biserial correlations indicated moderate-to-large paired effects for these comparisons.

Mean model ranks provide a complementary summary of relative performance. Lower ranks indicate better performance. Gradient Boosting achieved the best mean rank (1.98), followed by CatBoost (2.47), AdaBoost (2.91), XGBoost (3.53), and LightGBM (4.11). Gradient Boosting ranked highest for balanced accuracy and ROC-AUC, whereas CatBoost obtained the best F1-score rank. Thus, although Gradient Boosting had the strongest overall relative ranking, the analysis does not demonstrate that any single algorithm was the highest-performing model across all datasets and endpoints.

### 6.3. Explainability Analysis

SHAP and LIME were applied to disease-specific models selected exclusively from training-derived out-of-fold evidence, thereby avoiding selection of the explanation model based on held-out test performance. AdaBoost was selected for heart disease, CatBoost for lung cancer, and AdaBoost for diabetes. SHAP was used to characterize global patterns of feature contribution, while SHAP and LIME were used to examine representative individual predictions. All resulting explanations describe model behavior and should not be interpreted as evidence of causal clinical effects.

#### 6.3.1. Global SHAP Analysis

**Figure 19:**
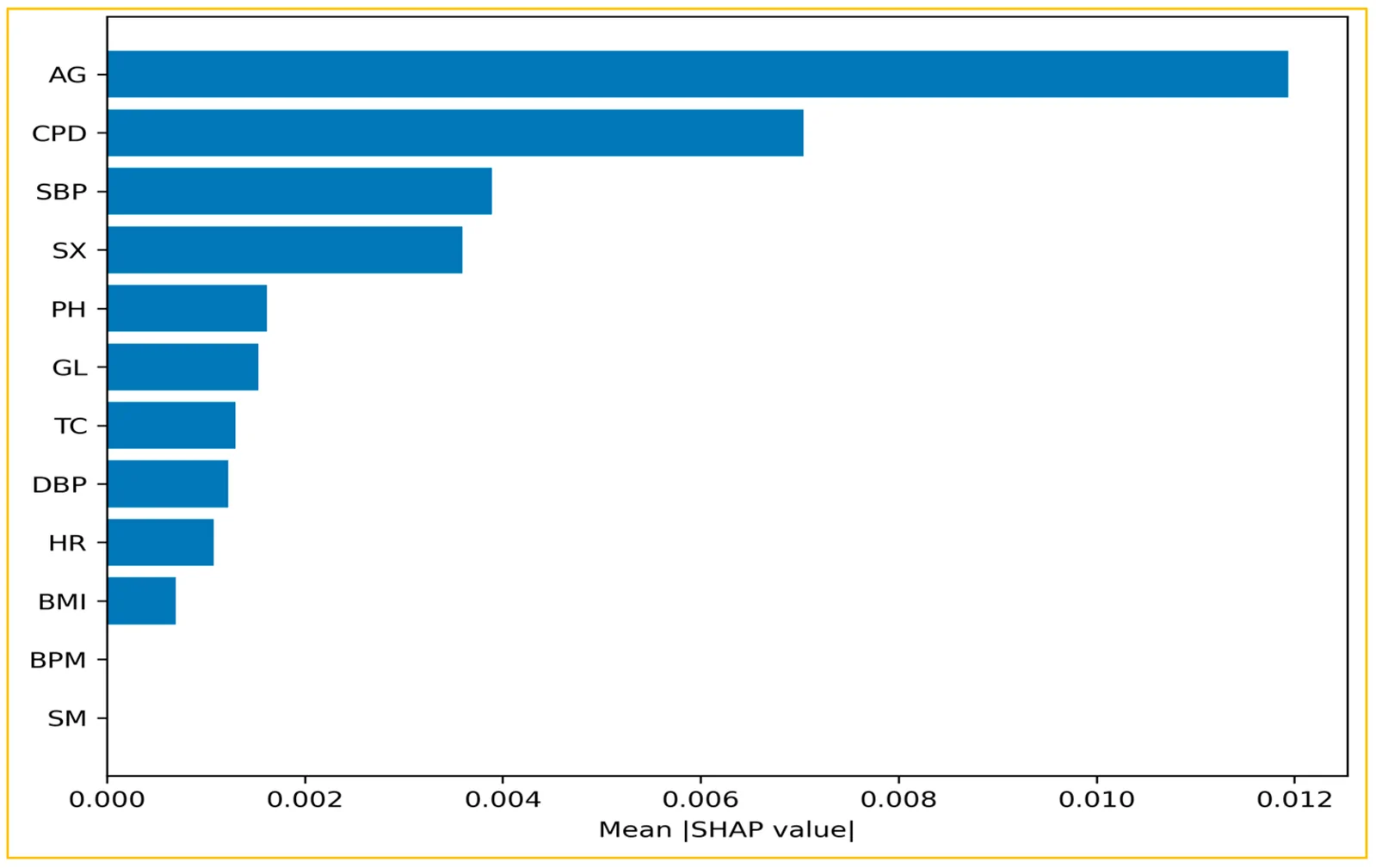
Global SHAP feature contributions for the AdaBoost heart disease model.

For heart disease, the AdaBoost SHAP analysis identified age (AG) as the largest global contributor, followed by cigarettes per day (CPD), systolic blood pressure (SBP), sex (SX), and prevalent hypertension (PH), as depicted in Figure 19. These rankings indicate which variables made the largest model-attributed contributions across the analyzed observations but do not establish that these variables causally determine heart disease.

**Figure 20:**
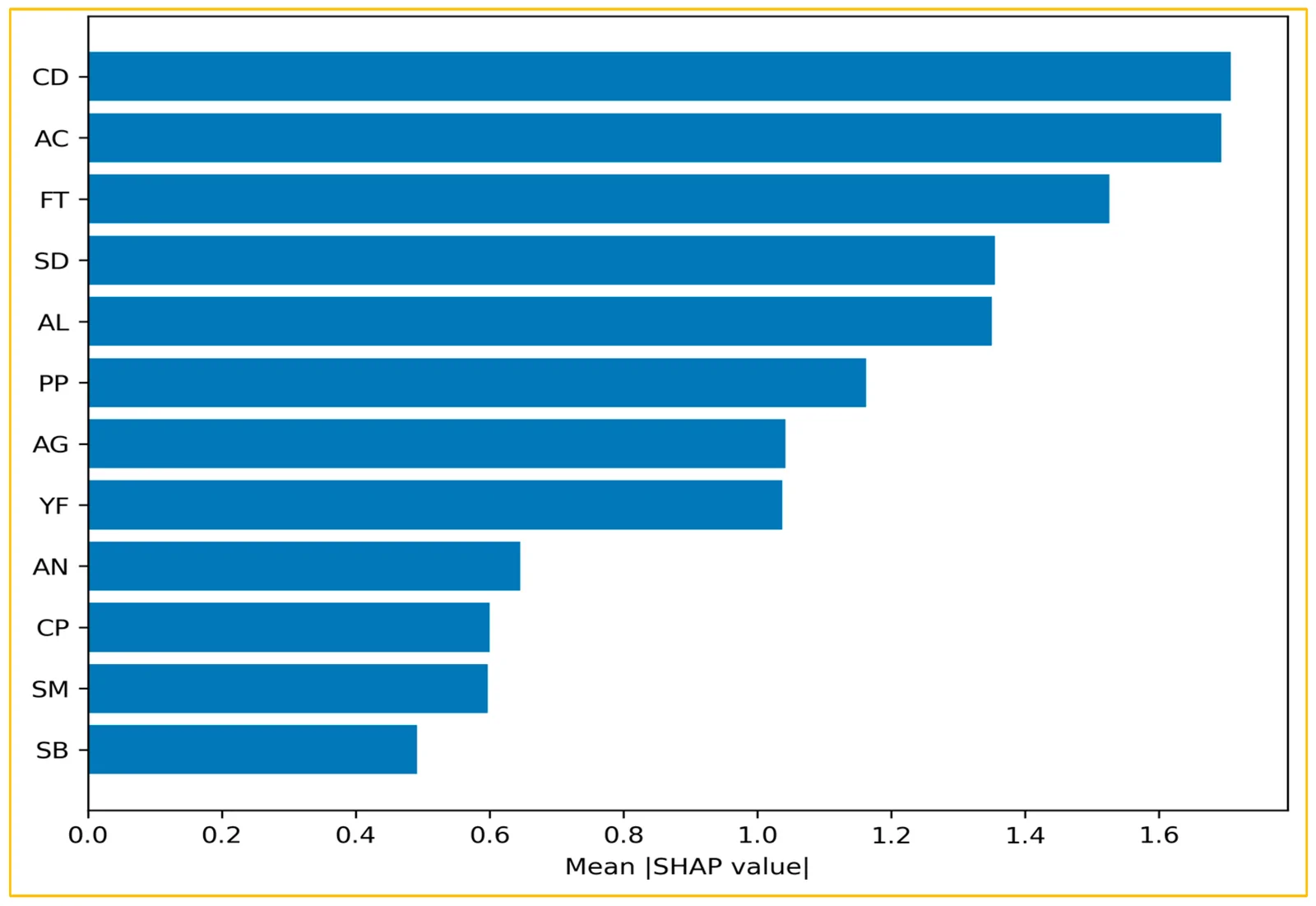
Global SHAP feature contributions for the CatBoost lung cancer model.

For lung cancer, the CatBoost explanation identified chronic disease (CD), alcohol consumption (AC), fatigue (FT), swallowing difficulty (SD), and allergy (AL) as the leading contributors, followed by peer pressure, age, and yellow fingers, as shown in Figure 20. Because this benchmark consists predominantly of self-reported symptom and lifestyle variables, these importance patterns should be regarded as characteristics of the fitted model and dataset rather than clinically validated risk-factor rankings.

**Figure 21:**
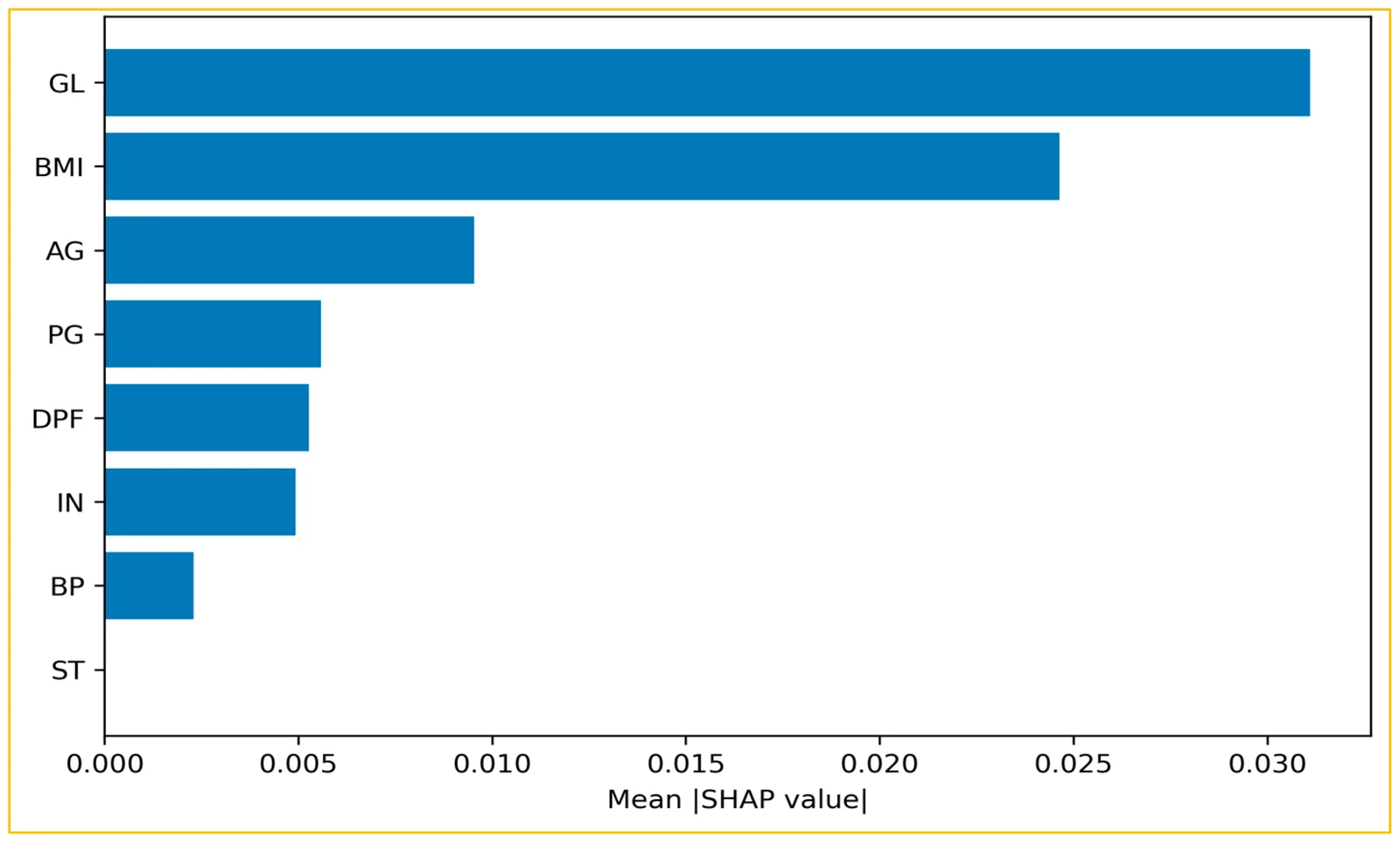
Global SHAP feature contributions for the AdaBoost diabetes model.

For diabetes, the AdaBoost SHAP analysis identified glucose (GL) and BMI as the two dominant contributors, followed by age (AG), pregnancies (PG), diabetes pedigree function (DPF), and insulin (IN), as illustrated in Figure 21. Skin thickness showed negligible global contribution in the fitted AdaBoost model. Again, these values quantify model-attributed feature contributions rather than causal disease contributions.

#### 6.3.2. Local LIME Analysis

Representative observations for local explanation were selected algorithmically rather than manually. Among correctly classified observations of each true class, the case with a calibrated probability closest to the corresponding class-specific median was selected. This approach reduces the risk of presenting only unusually confident or visually striking examples. LIME visualizations were then used to illustrate how individual features shifted each model output toward or away from the positive class. Because the decision thresholds were derived from training data and were not necessarily 0.50, the local probabilities were interpreted relative to the corresponding locked model-specific threshold.

**Figure 22:**
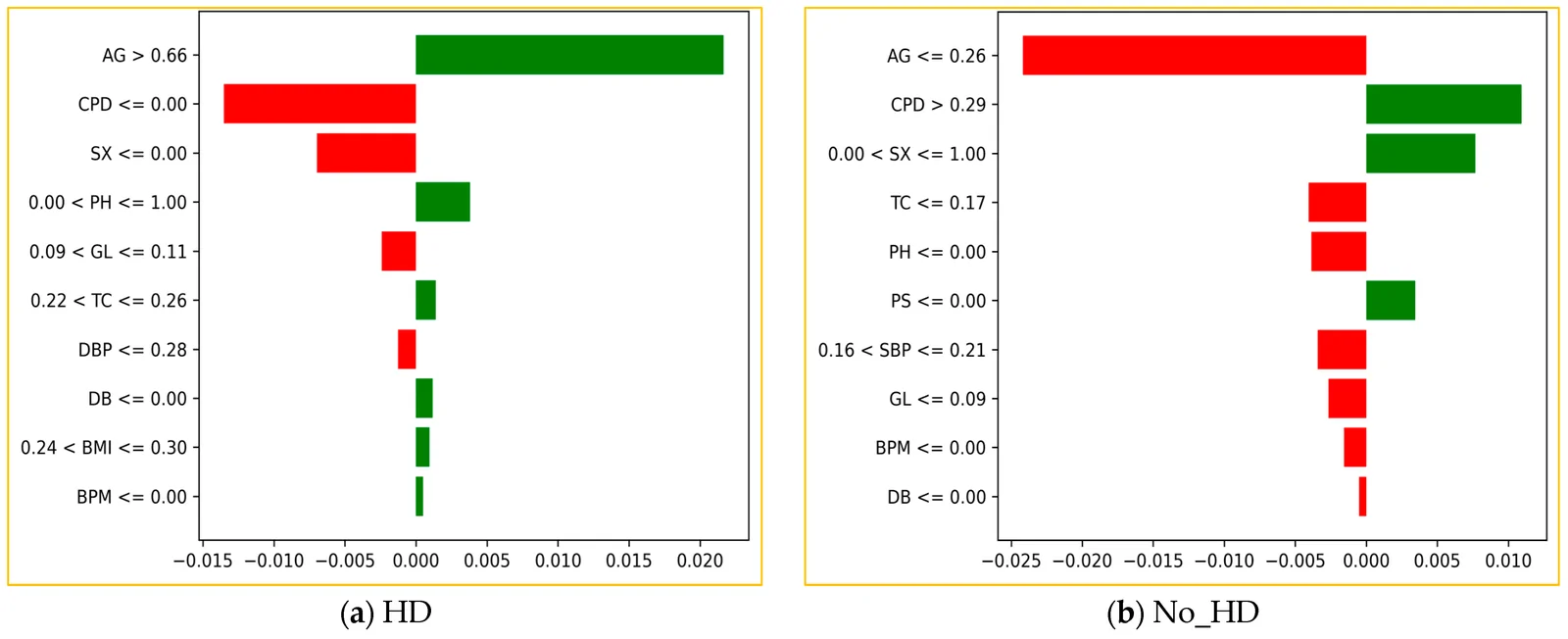
Representative local LIME (**a**) heart disease and (**b**) no heart disease explanations for the AdaBoost heart disease model.

**Figure 23:**
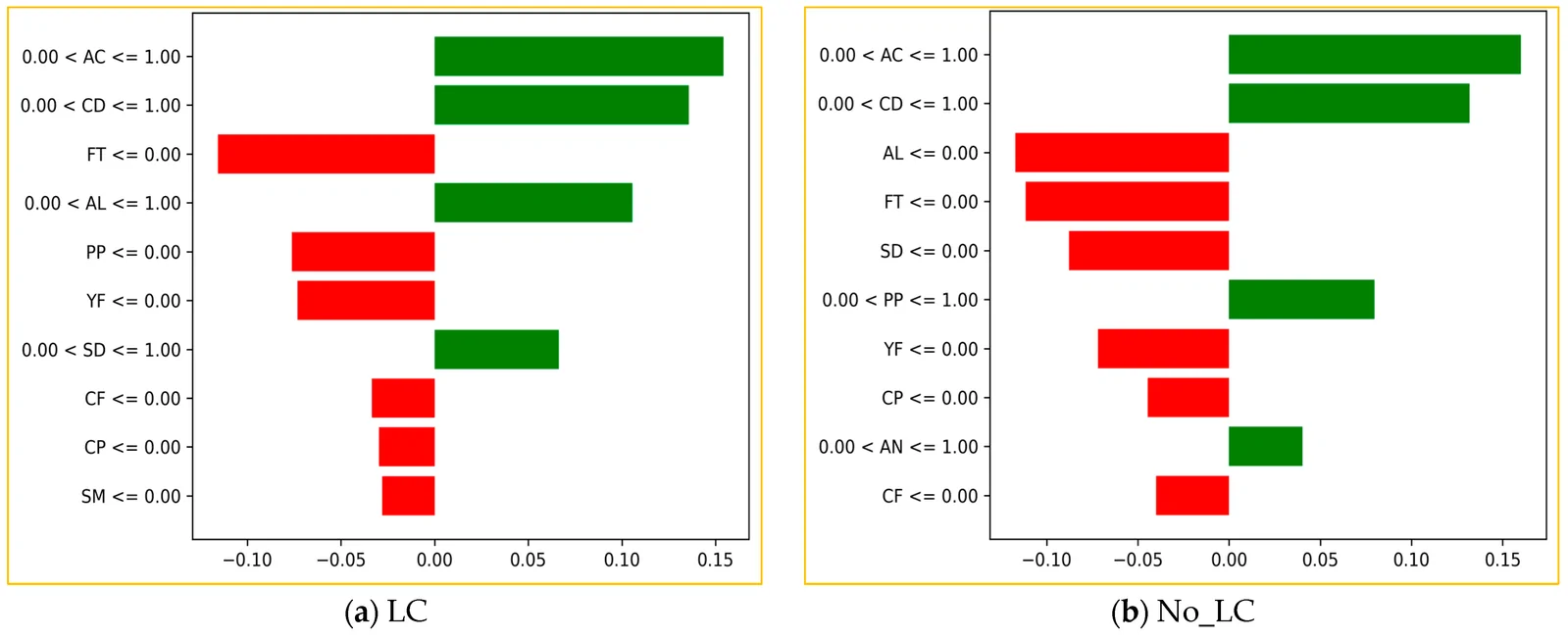
Representative local LIME (**a**) lung cancer and (**b**) no lung cancer explanations for the CatBoost lung cancer model.

**Figure 24:**
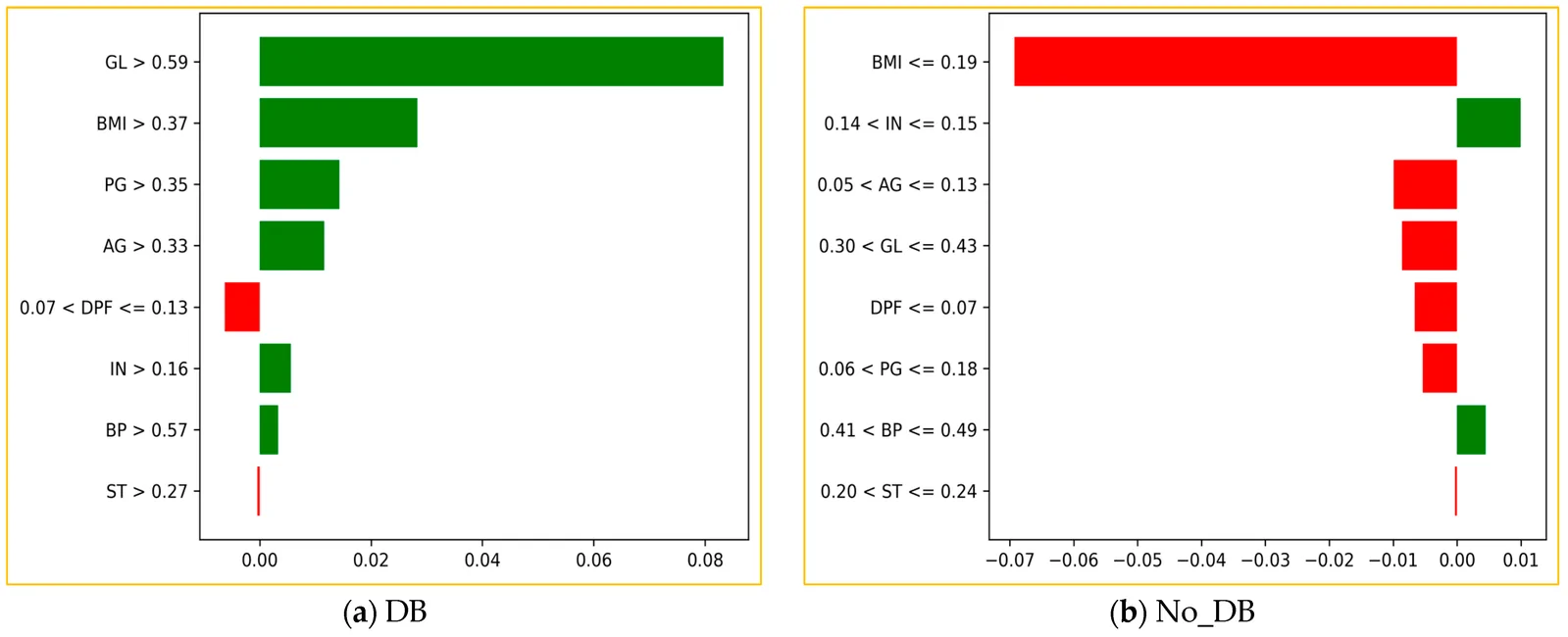
Representative local LIME (**a**) diabetes and (**b**) no diabetes explanations for the AdaBoost diabetes model.

The local explanations demonstrated that the contribution of individual variables varied across observations as shown in Figure 22, Figure 23 and Figure 24, even when the same final class was predicted. This reinforces the distinction between global importance and instance-specific explanation. Importantly, a positive LIME contribution indicates that a variable moved the model output toward the positive class for that particular observation; it does not imply that changing that variable would causally alter disease risk.

## 7. Discussion, Limitations, and Clinical Implications

This section discusses the principal findings from the leakage-free evaluation framework, with particular emphasis on differences across disease-specific tasks, model discrimination, class-sensitive performance, statistical comparisons, probability calibration, and explainability. The study should be interpreted as a comparative methodological benchmark across three independent chronic disease datasets rather than as an integrated patient-level multi-disease prediction system. The limitations of the datasets and experimental design are therefore considered alongside the potential future clinical relevance of explainable boosting-based disease-risk prediction.

### 7.1. Discussion and Limitations

The experiments demonstrate that the relative performance of boosting algorithms differs substantially across disease-specific datasets and evaluation endpoints. The present results were obtained after separating the held-out test data before fitted preprocessing, random oversampling, hyperparameter optimization, probability calibration, and threshold selection. Consequently, the resulting performance estimates are more conservative but provide a methodologically stronger assessment of model generalization. The findings also demonstrate the importance of evaluating imbalanced health datasets using complementary metrics rather than relying primarily on overall accuracy.

For heart disease, the differences among the five boosting algorithms were comparatively small. AdaBoost achieved the highest balanced accuracy (68.07%), sensitivity (67.96%), F1-score (39.33%), and ROC-AUC (71.71%), whereas LightGBM obtained the highest overall accuracy (70.35%) and specificity (72.00%). The relatively low precision and F1-scores observed across all models reflect the substantial imbalance in the heart disease dataset, in which positive cases constituted only about 15% of the observations. Importantly, the use of the conventional 0.50 probability threshold resulted in very low sensitivity for several models, whereas the thresholds selected exclusively from training-derived out-of-fold probabilities produced a more balanced relationship between sensitivity and specificity. This finding illustrates that a high raw accuracy can be misleading in imbalanced disease-prediction tasks when a model predominantly favors the majority class.

The lung cancer dataset produced substantially stronger discrimination than the heart disease dataset. CatBoost achieved the highest balanced accuracy (92.59%), ROC-AUC (96.30%), and F1-score (92.00%), while LightGBM showed closely comparable performance. However, these results require particular caution because the held-out test set contained only eight lung-cancer-negative observations. Although all five models achieved 100% specificity at their locked thresholds, this corresponds to correctly classifying only eight negative cases and should not be interpreted as evidence of perfect specificity in a broader clinical population. Furthermore, the positive class constituted the large majority of the dataset, which also contributed to the very high PR-AUC values. Therefore, the apparently strong lung cancer performance is best interpreted as internal benchmark evidence rather than an estimate of prospective diagnostic performance.

For diabetes, model differences were again relatively modest. XGBoost achieved the highest balanced accuracy (77.02%) and sensitivity (87.04%), whereas Gradient Boosting achieved the highest ROC-AUC (82.43%) and PR-AUC (69.70%) together with the lowest Brier score (0.1614). CatBoost exhibited higher specificity but lower sensitivity than XGBoost. These differences demonstrate that the preferred algorithm depends on the intended evaluation objective. A model that maximizes case detection may produce more false-positive predictions, whereas a model with higher specificity may fail to identify additional positive cases. Therefore, no single model can be considered optimal solely on the basis of one metric.

The repeated cross-validation analysis provides complementary evidence beyond the individual held-out test sets. The Friedman tests identified significant overall differences among the algorithms for balanced accuracy, ROC-AUC, and F1-score. Across the matched dataset–repeat blocks, Gradient Boosting achieved the best mean rank (1.98), followed by CatBoost (2.47), AdaBoost (2.91), XGBoost (3.53), and LightGBM (4.11). However, the post hoc analysis did not indicate that Gradient Boosting was the significantly highest-performing model compared to every competing algorithm for every endpoint. CatBoost achieved the best mean rank for F1-score, while significant pairwise differences varied by the metric under consideration. These results therefore support the conclusion that algorithm performance is endpoint- and dataset-dependent rather than demonstrating universal superiority of a single boosting technique. Because the 15 matched dataset–repeat blocks were derived from only three underlying datasets and observations generated through repeated cross-validation remain partially correlated, the associated inferential tests should be regarded as supportive internal comparative evidence rather than independent replication or a substitute for external validation.

Probability calibration provides an additional perspective that is particularly relevant when model outputs are interpreted as risk estimates. Brier scores were incorporated together with ROC-AUC and PR-AUC so that model assessment was not limited to classification discrimination. Across the held-out datasets, models with similar ROC-AUC values sometimes exhibited different Brier scores and threshold-dependent behavior. This distinction is important because strong discrimination does not necessarily imply that predicted probabilities are well calibrated. Nevertheless, the calibration procedures in this study were evaluated only internally, and calibration should be reassessed and, where necessary, updated before any application to populations with different disease prevalence or demographic characteristics.

The explainability analysis further demonstrated that the models relied on different combinations of demographic, lifestyle, symptom-related, and physiological variables across the three prediction tasks. Importantly, the models selected for detailed XAI analysis were determined from training-only evidence rather than from their held-out test results: AdaBoost was used for heart disease, CatBoost for lung cancer, and AdaBoost for diabetes. SHAP provided global information regarding the relative contribution of individual variables, whereas local SHAP and LIME explanations illustrated how specific feature values shifted individual predictions. These analyses improve transparency of the fitted models but do not establish causal relationships. A feature receiving a large SHAP value should therefore be interpreted as strongly contributing to the model output under the observed data distribution rather than as evidence that modifying that feature would necessarily alter disease risk.

Several limitations should be considered when interpreting these findings. First, all three datasets were obtained from publicly available repositories and differ substantially in sample size, class prevalence, feature composition, data-collection context, and clinical representativeness. Consequently, performance differences across the three tasks should not be attributed solely to the algorithms themselves. Second, the lung cancer dataset is particularly small and highly imbalanced, with only 309 total observations and 39 negative cases; therefore, estimates of specificity and other class-dependent measures are subject to substantial uncertainty. Third, the datasets are independent and do not contain the same individuals across heart disease, lung cancer, and diabetes. The present study therefore evaluates three independent binary prediction tasks and does not estimate patient-level multimorbidity, disease co-occurrence, or an aggregated multi-disease risk score.

Fourth, missing-value treatment introduces assumptions into the modeling process. Median imputation was selected as a reproducible and comparatively robust strategy, but any single-value imputation method may reduce variability and does not account for uncertainty associated with missing observations. For the diabetes dataset, physiologically implausible zero values in selected variables were treated as missing within the modeling pipeline; although this is preferable to treating such measurements as physiologically meaningful, it remains dependent on the structure and coding of the benchmark dataset. Fifth, random oversampling was used within training folds to address class imbalance. Although this avoids leakage into validation and test observations, duplicated minority-class records do not provide additional biological or clinical diversity and may still influence decision boundaries.

Sixth, model development and final assessment were based on an internal train–test split and repeated cross-validation; no independent external cohort, temporal validation cohort, or prospective clinical dataset was available. The reported estimates therefore reflect internal benchmark performance and cannot establish transportability across hospitals, geographic regions, demographic groups, or disease-prevalence settings. Seventh, subgroup performance according to demographic or clinically relevant strata was not comprehensively evaluated. Potential differences in error rates across sex, age, or other population subgroups should therefore be investigated before considering any real-world deployment.

Finally, SHAP and LIME themselves have methodological limitations. SHAP values can be influenced by correlations among predictors and by the choice of background distribution, whereas LIME explanations depend on local perturbations and may vary across repeated runs or parameter settings. Representative local cases were selected algorithmically in the analysis to reduce cherry-picking of extreme predictions, but these examples still cannot establish the stability of explanations across all individuals. Future studies should therefore investigate explanation stability, subgroup consistency, external calibration, and prospective generalizability using larger and clinically curated datasets.

### 7.2. Clinical Implications and Applicability

The findings of this study should primarily be interpreted as evidence of the methodological feasibility of applying a common leakage-aware, calibrated, and explainable boosting framework across heterogeneous disease-specific datasets. The models were not developed or evaluated as autonomous diagnostic systems, and their predictions should not be interpreted as replacements for clinical assessment. Instead, the present analysis demonstrates how established boosting algorithms can be compared under a reproducible protocol that integrates class-imbalance handling, probability calibration, threshold optimization, uncertainty estimation, repeated cross-validation, statistical comparison, and model explanation.

From a future clinical perspective, the simultaneous consideration of discrimination, sensitivity, specificity, calibration, and explanation is more informative than reporting accuracy alone. For example, the heart disease results demonstrate that a classifier can achieve comparatively high apparent accuracy while providing inadequate sensitivity at a conventional threshold. In a clinical risk-screening setting, such behavior could result in failure to identify individuals who may require additional assessment. Conversely, lowering a threshold to improve sensitivity can increase false-positive predictions and potentially lead to unnecessary follow-up. The appropriate operating threshold would therefore need to be selected according to the intended clinical context, relative consequences of false-negative and false-positive decisions, disease prevalence, and available healthcare resources rather than being adopted directly from the present benchmark.

Probability calibration would also be necessary if predicted outputs were to be communicated as individualized risk estimates. Well-calibrated probabilities may potentially support risk stratification, but the calibration observed in the present datasets cannot be assumed to transfer directly to other populations. External recalibration and prospective validation would be required before numerical probabilities could be used to guide screening, referral, or treatment decisions.

The integration of SHAP and LIME illustrates how model-specific predictions can be accompanied by information regarding the variables contributing to those predictions. In a future externally validated decision-support system, such explanations could potentially assist clinicians in examining whether a prediction is consistent with the available patient information and identifying which variables exerted the greatest influence on the algorithmic output. However, these explanations should be presented as model-attribution information rather than clinical or causal evidence. For example, a positive SHAP contribution indicates that a feature shifted the fitted model toward the disease class; it does not demonstrate that the feature caused the disease or that modifying it would necessarily reduce an individual’s risk.

The three disease models should also remain clinically distinct. Because the heart disease, lung cancer, and diabetes datasets contain different individuals, variables, and outcome definitions, their predictions cannot currently be combined into an integrated patient-level chronic disease score. A clinically meaningful multi-disease risk system would require a common longitudinal cohort in which the same individuals are characterized for multiple outcomes, preferably with standardized demographic, lifestyle, laboratory, and clinical variables and appropriate follow-up. Such data would permit investigation of disease co-occurrence, shared risk factors, temporal relationships, and patient-level multimorbidity, none of which can be established using the present independent datasets.

Accordingly, several stages would be required before any practical clinical application: validation using larger and demographically diverse multicenter cohorts; evaluation of discrimination and calibration across relevant patient subgroups; prospective assessment under realistic disease prevalence; investigation of model and explanation stability; comparison with existing clinical risk scores where available; and evaluation of how algorithmic predictions affect clinician decisions, patient outcomes, resource utilization, and workflow. Until these steps are completed, the current models should be regarded as research benchmarks rather than clinically deployable decision-support tools.

## 8. Comparative Analysis

This section synthesizes the comparative behavior of the five boosting algorithms across the three disease-specific prediction tasks and contextualizes the findings with selected previous studies. Because the heart disease, lung cancer, and diabetes datasets differ substantially in sample size, class prevalence, predictor composition, and clinical context, comparisons across datasets should be interpreted as benchmarking evidence rather than as direct estimates of relative difficulty in disease prediction. Similarly, comparisons with previously published studies should be treated as descriptive, as differences in datasets, preprocessing, validation procedures, class-balancing methods, evaluation thresholds, and reported metrics preclude direct statistical comparison.

### 8.1. Comparison Among the Evaluated Boosting Models

The comparative results presented in Table 10 indicate that no single boosting algorithm was the highest-performing model across all disease datasets and evaluation measures. On the held-out heart disease test set, AdaBoost achieved the highest balanced accuracy (68.07%), sensitivity (67.96%), F1-score (39.33%), and ROC-AUC (71.71%), whereas LightGBM achieved the highest overall accuracy (70.35%) and specificity (72.00%). For lung cancer, CatBoost achieved the highest held-out balanced accuracy (92.59%), F1-score (92.00%), and ROC-AUC (96.30%) among the evaluated models. For diabetes, XGBoost achieved the highest balanced accuracy (77.02%) and sensitivity (87.04%), whereas Gradient Boosting achieved the highest ROC-AUC (82.43%), PR-AUC (69.70%), and the lowest Brier score (0.1614).

These disease-specific results demonstrate that model ranking depends strongly on the selected endpoint. For example, emphasizing sensitivity would favor XGBoost for diabetes, whereas emphasizing discrimination or probabilistic performance would favor Gradient Boosting. Similarly, in the heart disease task, the model with the highest accuracy was not the model with the highest balanced accuracy or sensitivity. This finding is particularly relevant for imbalanced healthcare datasets, where accuracy may obscure clinically important false-negative and false-positive behavior.

The supplementary repeated cross-validation analysis provides a broader comparison across datasets. Gradient Boosting obtained the best mean rank of 1.98, followed by CatBoost at 2.47, AdaBoost at 2.91, XGBoost at 3.53, and LightGBM at 4.11. However, the endpoint-specific ranking differed: Gradient Boosting ranked first for balanced accuracy and ROC-AUC, whereas CatBoost ranked first for F1-score. The Friedman and Holm-adjusted Wilcoxon analyses further showed that statistically significant pairwise differences depended on the evaluation endpoint. Also, Table 11 presents the contributions of the top predicted variables, as identified by SHAP analysis, across datasets for the considered models. Therefore, the comparative evidence supports model-specific strengths rather than universal dominance by one algorithm.

### 8.2. Comparison with the Existing Similar Works

Previous studies have reported machine-learning prediction of heart disease, lung cancer, and diabetes using logistic regression, boosting algorithms, and other ensemble methods [25,26,27,28,31,33,55,56,57,58,59,60,61]. However, direct numerical comparison with earlier studies is not straightforward because studies differ in dataset size, disease prevalence, predictors, preprocessing, class-imbalance handling, model selection, cross-validation, and evaluation metrics. Thus, higher or lower accuracy, F1-score, or AUC across studies does not necessarily indicate better or worse performance. The present study therefore emphasizes methodological consistency across three prediction tasks rather than that one model was universally superior. All five boosting algorithms were evaluated in a leakage-free framework: held-out test sets were separated before preprocessing and resampling, class balancing was limited to training folds, hyperparameters were optimized by stratified cross-validation, and calibration and threshold selection were performed without test labels.

Performance was characterized using discrimination and calibration measures with confidence intervals. The results also show why accuracy alone may mislead. For heart disease, the highest-accuracy model did not achieve the highest sensitivity, balanced accuracy, F1-score, or ROC-AUC. For diabetes, the preferred model depended on whether balanced accuracy, sensitivity, discrimination, or probabilistic performance was emphasized. For lung cancer, discrimination was strong, but interpretation is constrained by few negative observations in the held-out test set. These findings highlight the importance of class distribution, uncertainty, calibration, and error characteristics when evaluating disease-prediction models. The study provides a reproducible internal benchmark of established boosting algorithms across heterogeneous chronic disease datasets. Independent external validation using comparable populations and evaluation protocols would be required before claims of comparative clinical superiority could be established.

## 9. Conclusions and Future Scope

This study presented a common explainable boosting framework for comparative disease-specific prediction across three independent datasets representing heart disease, lung cancer, and diabetes. Five established boosting algorithms, XGBoost, CatBoost, LightGBM, AdaBoost, and Gradient Boosting, were evaluated under a common leakage-free experimental protocol. The workflow separated the held-out test data before fitted preprocessing and class balancing, restricted hyperparameter optimization and probability calibration to the training data, selected decision thresholds from training-only out-of-fold predictions, and evaluated final performance using multiple complementary discrimination, classification, calibration, and uncertainty measures.

The held-out results demonstrated that no single boosting algorithm was consistently superior across all disease datasets and evaluation criteria. For heart disease, AdaBoost achieved the highest balanced accuracy of 68.07% and ROC-AUC of 71.71%, whereas LightGBM achieved the highest overall accuracy of 70.35%. For lung cancer, CatBoost achieved the strongest overall held-out performance, with a balanced accuracy of 92.59% and ROC-AUC of 96.30%; however, this result should be interpreted cautiously because the test set contained only eight lung-cancer-negative observations. For diabetes, XGBoost achieved the highest balanced accuracy of 77.02%, while Gradient Boosting achieved the highest ROC-AUC of 82.43% and the lowest Brier score of 0.1614.

The repeated cross-validation analysis provided additional evidence of relative model performance. Significant overall differences were identified among the five algorithms for balanced accuracy, ROC-AUC, and F1-score. Gradient Boosting achieved the best mean rank (1.98), followed by CatBoost (2.47), although the endpoint-specific rankings and post hoc comparisons showed that no model was uniformly superior across all evaluation measures. These findings reinforce the importance of evaluating machine-learning models using multiple complementary endpoints rather than relying on accuracy alone.

The incorporation of SHAP and LIME further improved transparency by characterizing global and local feature contributions for disease-specific models selected exclusively from training-derived evidence. AdaBoost was selected for heart disease and diabetes, while CatBoost was selected for lung cancer. The explainability analyses identified variables with substantial model-attributed contributions; however, these contributions represent characteristics of the fitted models and observed data rather than causal clinical relationships and should not be interpreted as evidence that modifying a particular variable would necessarily change disease risk.

Overall, the findings demonstrate the feasibility of applying a consistent, leakage-free, calibrated, statistically evaluated, and explainable boosting workflow across heterogeneous chronic disease datasets. At the same time, the results should be regarded as internal benchmark evidence rather than evidence of clinical readiness. The three datasets contain different individuals, predictor sets, and outcome definitions; therefore, the present study does not estimate patient-level multimorbidity or an integrated multi-disease risk score. Furthermore, the reliance on publicly available datasets, limited sample sizes, class imbalance, absence of external validation, and potential instability of local explanations restrict the generalizability of the current findings.

Future work should therefore prioritize validation using larger, multicenter, demographically diverse, and prospectively collected clinical cohorts. External and temporal validation should be performed to assess model transportability, while subgroup analyses should examine whether discrimination, calibration, and error rates differ across age, sex, and other clinically relevant population groups. Future studies should also investigate more advanced missing-data strategies, cost-sensitive learning, alternative imbalance-handling methods, and external recalibration under different disease-prevalence settings. The stability and reproducibility of SHAP and LIME explanations should additionally be assessed across repeated model fits and independent cohorts.

A further important direction is the development of genuine multi-disease prediction systems using a common patient cohort in which the same individuals are characterized for multiple chronic conditions over time. Such data would permit investigation of disease co-occurrence, shared risk factors, longitudinal trajectories, and multimorbidity interactions that cannot be evaluated using the independent datasets employed in the present study. Future work may also explore multimodal integration of structured clinical variables with laboratory, imaging, genomic, wearable-sensor, and longitudinal electronic health-record data. Following rigorous external and prospective validation, appropriately calibrated and explainable models could then be investigated for potential integration into clinical decision-support or digital-health environments.

## Figures and Tables

**Table 1:** Hyperparameter search spaces used for the five boosting algorithms in the experiments.

Model	Hyperparameter	Heart Disease	Lung Cancer	Diabetes
XGBoost	n_estimators	300, 600, 1000	200, 500, 800	200, 500, 800
	learning_rate	0.02, 0.05, 0.10	0.02, 0.05, 0.10	0.02, 0.05, 0.10
	max_depth	3, 5, 7	2, 3, 5	2, 3, 5
	min_child_weight	1, 3, 5	1, 3	1, 3, 5
	subsample	0.8, 1.0	0.8, 1.0	0.8, 1.0
	colsample_bytree	0.8, 1.0	0.8, 1.0	0.8, 1.0
CatBoost	iterations	300, 600, 1000	200, 500, 800	200, 500, 800
	learning_rate	0.02, 0.05, 0.10	0.02, 0.05, 0.10	0.02, 0.05, 0.10
	depth	4, 6, 8	3, 5, 7	3, 5, 7
	l2_leaf_reg	3, 7, 12	3, 7, 12	3, 7, 12
LightGBM	n_estimators	300, 600, 1000	200, 500, 800	200, 500, 800
	learning_rate	0.02, 0.05, 0.10	0.02, 0.05, 0.10	0.02, 0.05, 0.10
	max_depth	3, 5, 7	2, 3, 5	2, 3, 5
	num_leaves	15, 31, 63	7, 15, 31	7, 15, 31
	min_child_samples	20, 50	10, 20, 30	10, 20, 30
AdaBoost	n_estimators	100, 300, 600	100, 300, 500	100, 300, 500
	learning_rate	0.02, 0.05, 0.10, 0.50	0.02, 0.05, 0.10, 0.50	0.02, 0.05, 0.10, 0.50
Gradient Boosting	n_estimators	200, 400, 600	100, 300, 500	100, 300, 500
	learning_rate	0.02, 0.05, 0.10	0.02, 0.05, 0.10	0.02, 0.05, 0.10
	max_depth	2, 3, 5	1, 2, 3	1, 2, 3
	min_samples_leaf	1, 5, 10	1, 3, 5	1, 3, 5, 10
	subsample	0.8, 1.0	0.8, 1.0	0.8, 1.0

**Table 2:** Hardware and software environment used for the experiments.

Component	Specification
Processor	Intel^®^ Core™ i9-10900K CPU @ 3.70 GHz
RAM	64 GB DDR4
Primary storage	500 GB NVMe SSD
Secondary storage	2 TB HDD
Operating system	Windows 11 Pro
Programming language	Python
Development environment	Jupyter Notebook
Machine-learning framework	scikit-learn
Class-imbalance handling	imbalanced-learn
Boosting libraries	XGBoost, CatBoost, LightGBM, scikit-learn
Explainability libraries	SHAP and LIME

**Table 3:** Summary of the three independent disease-specific datasets used in this study.

Dataset	Samples	Variables	Key Predictors Included	Outcome
Heart Disease	3390	15	Age, sex, BMI, systolic blood pressure, diastolic blood pressure, total cholesterol, glucose, heart rate, cigarettes per day, prevalent hypertension, prevalent stroke, diabetes	Heart disease status
Lung Cancer	309	16	Age, gender, smoking, yellow fingers, anxiety, peer pressure, chronic disease, fatigue, allergy, wheezing, alcohol consumption, coughing, shortness of breath, swallowing difficulty, chest pain	Lung cancer status
Diabetes	768	9	Pregnancies, glucose, blood pressure, skin thickness, insulin, BMI, diabetes pedigree function, age	Diabetes status

**Table 4:** Performance of boosting models on the heart disease test set using training-derived decision thresholds.

Model	(95% CI)								
	Accuracy	Balanced Accuracy	Precision	Sensitivity	Specificity	F1-Score	ROC-AUC	PR-AUC	Brier Score
XGBoost	69.32 (65.8–72.7)	65.58 (60.4–70.7)	27.07 (21.5–32.9)	60.19 (50.6–70.0)	70.96 (67.2–74.9)	37.35 (30.6–43.9)	71.36 (65.3–76.8)	33.29 (25.9–42.5)	0.1183 (0.102–0.137)
CatBoost	68.88 (65.3–72.3)	66.91 (61.7–72.0)	27.50 (21.9–33.3)	64.08 (54.4–73.5)	69.74 (65.9–73.6)	38.48 (31.7–44.9)	71.20 (65.3–76.7)	33.88 (26.2–42.6)	0.1185 (0.102–0.137)
LightGBM	70.35 (67.0–73.7)	66.58 (61.6–71.7)	28.13 (22.6–34.1)	61.17 (51.3–70.6)	72.00 (68.4–75.7)	38.53 (31.7–45.2)	71.34 (65.4–76.8)	32.07 (25.2–41.8)	0.1185 (0.102–0.137)
AdaBoost	68.14 (64.7–71.7)	68.07 (63.0–72.8)	27.67 (22.5–33.2)	67.96 (58.3–76.6)	68.17 (64.3–72.0)	39.33 (32.9–45.4)	71.71 (66.0–76.8)	33.43 (26.3–42.9)	0.1177 (0.101–0.136)
Gradient Boosting	68.73 (65.3–72.3)	67.62 (62.5–72.5)	27.76 (22.5–33.3)	66.02 (56.7–75.3)	69.22 (65.4–73.1)	39.08 (32.4–45.5)	70.92 (65.0–76.4)	33.08 (25.7–42.1)	0.1183 (0.101–0.137)

**Table 5:** Performance of boosting models on the held-out lung cancer test set using training-derived decision thresholds.

Model	(95% CI)								
	Accuracy	Balanced Accuracy	Precision	Sensitivity	Specificity	F1-Score	ROC-AUC	PR-AUC	Brier Score
XGBoost	82.26 (72.6–91.9)	89.81 (84.3–95.0)	100.00 (100.0–100.0)	79.63 (68.5–90.0)	100.00 (100.0–100.0)	88.66 (81.3–94.7)	93.75 (86.3–99.2)	99.08 (97.7–99.9)	0.0737 (0.037–0.115)
CatBoost	87.10 (77.4–95.2)	92.59 (87.5–97.1)	100.00 (100.0–100.0)	85.19 (75.0–94.1)	100.00 (100.0–100.0)	92.00 (85.7–97.0)	96.30 (90.6–99.7)	99.47 (98.5–100.0)	0.0593 (0.030–0.094)
LightGBM	85.48 (75.8–93.5)	91.67 (86.4–96.4)	100.00 (100.0–100.0)	83.33 (72.7–92.7)	100.00 (100.0–100.0)	90.91 (84.2–96.2)	96.06 (90.2–99.7)	99.43 (98.5–100.0)	0.0612 (0.029–0.100)
AdaBoost	72.58 (61.3–82.3)	84.26 (77.7–90.2)	100.00 (100.0–100.0)	68.52 (55.4–80.4)	100.00 (100.0–100.0)	81.32 (71.3–89.1)	93.17 (84.7–98.9)	98.97 (97.4–99.9)	0.0766 (0.043–0.118)
Gradient Boosting	79.03 (69.4–88.7)	87.96 (82.0–93.4)	100.00 (100.0–100.0)	75.93 (64.0–86.8)	100.00 (100.0–100.0)	86.32 (78.0–92.9)	93.98 (85.8–99.3)	99.10 (97.6–99.9)	0.0640 (0.034–0.101)

**Table 6:** Performance of boosting models on the held-out diabetes test set using training-derived decision thresholds.

Model	(95% CI)								
	Accuracy	Balanced Accuracy	Precision	Sensitivity	Specificity	F1-Score	ROC-AUC	PR-AUC	Brier Score
XGBoost	74.03 (66.9–80.5)	77.02 (70.4–83.2)	58.75 (47.9–69.1)	87.04 (77.6–95.9)	67.00 (57.6–75.8)	70.15 (60.7–78.4)	81.93 (74.7–88.2)	68.00 (55.0–80.3)	0.1645 (0.134–0.199)
CatBoost	74.68 (68.2–81.2)	74.54 (67.5–81.5)	61.54 (50.0–73.1)	74.07 (62.7–85.7)	75.00 (66.3–83.2)	67.23 (57.1–76.3)	81.74 (74.5–88.0)	67.14 (54.0–80.0)	0.1670 (0.135–0.203)
LightGBM	74.68 (67.5–81.2)	76.67 (69.7–83.1)	60.00 (48.7–70.8)	83.33 (73.2–93.2)	70.00 (60.4–78.5)	69.77 (60.2–78.2)	82.15 (75.0–88.3)	66.52 (53.4–79.5)	0.1634 (0.133–0.198)
AdaBoost	74.68 (67.5–81.2)	74.96 (67.9–82.0)	61.19 (50.0–72.7)	75.93 (64.7–87.1)	74.00 (65.0–82.4)	67.77 (57.7–76.7)	82.38 (75.3–88.5)	67.75 (54.5–80.3)	0.1635 (0.134–0.196)
Gradient Boosting	73.38 (66.2–79.9)	75.67 (68.7–82.1)	58.44 (47.3–69.3)	83.33 (73.2–93.2)	68.00 (58.2–76.8)	68.70 (59.0–77.2)	82.43 (75.1–88.7)	69.70 (56.3–81.9)	0.1614 (0.131–0.197)

**Table 7:** Friedman tests across 15 matched dataset–repeat blocks.

Endpoint	$\chi^{2}$	df	*p*-Value	Interpretation
Balanced accuracy	26.293	4	0.000028	Significant
ROC-AUC	21.173	4	0.000293	Significant
F1-score	15.093	4	0.004512	Significant

**Table 8:** Mean model ranks across the 15 matched dataset–repeat blocks; lower ranks indicate better performance.

Model	Balanced Accuracy Rank	ROC-AUC Rank	F1 Rank	Overall Mean Rank
Gradient Boosting	**1.67**	**1.80**	2.47	**1.98**
CatBoost	2.73	2.80	**1.87**	2.47
AdaBoost	2.53	2.67	3.53	2.91
XGBoost	3.73	3.40	3.47	3.53
LightGBM	4.33	4.33	3.67	4.11

**Table 9:** Statistically significant pairwise Wilcoxon signed-rank comparisons after Holm correction.

Endpoint	Comparison	Holm-Adjusted *p*	Rank-Biserial r	Direction
Balanced accuracy	AdaBoost vs. LightGBM	0.0342	0.800	AdaBoost > LightGBM
Balanced accuracy	GB vs. LightGBM	0.00061	1.000	GB > LightGBM
Balanced accuracy	GB vs. XGBoost	0.00110	0.983	GB > XGBoost
ROC-AUC	GB vs. LightGBM	0.00061	1.000	GB > LightGBM
ROC-AUC	GB vs. XGBoost	0.00110	0.983	GB > XGBoost
ROC-AUC	XGBoost vs. LightGBM	0.0122	0.867	XGBoost > LightGBM
F1-score	CatBoost vs. AdaBoost	0.0201	0.850	CatBoost > AdaBoost
F1-score	GB vs. AdaBoost	0.0376	0.783	GB > AdaBoost
F1-score	CatBoost vs. LightGBM	0.0269	0.817	CatBoost > LightGBM
F1-score	CatBoost vs. XGBoost	0.0201	0.850	CatBoost > XGBoost

**Table 10:** Best-performing models on the held-out test sets according to complementary evaluation endpoints.

Dataset	Highest Accuracy	Highest Balanced Accuracy	Highest Sensitivity	Highest Specificity	Highest F1-Score	Highest ROC-AUC	Lowest Brier Score
Heart disease	LightGBM, 70.35%	AdaBoost, 68.07%	AdaBoost, 67.96%	LightGBM, 72.00%	AdaBoost, 39.33%	AdaBoost, 71.71%	AdaBoost, 0.1177
Lung cancer	CatBoost, 87.10%	CatBoost, 92.59%	CatBoost, 85.19%	All models, 100%	CatBoost, 92.00%	CatBoost, 96.30%	CatBoost, 0.0593
Diabetes	CatBoost/LightGBM/AdaBoost, 74.68%	XGBoost, 77.02%	XGBoost, 87.04%	CatBoost, 75.00%	XGBoost, 70.15%	Gradient Boosting, 82.43%	Gradient Boosting, 0.1614

**Table 11:** Leading global SHAP contributors for the disease-specific models selected using training-only evidence.

Dataset	XAI Model	Leading SHAP Contributors
Heart disease	AdaBoost	Age (AG), Cigarettes per Day (CPD), Systolic Blood Pressure (SBP), Sex (SX), Prevalent Hypertension (PH)
Lung cancer	CatBoost	Chronic Disease (CD), Alcohol Consumption (AC), Fatigue (FT), Swallowing Difficulty (SD), Allergy (AL)
Diabetes	AdaBoost	Glucose (GL), BMI, Age (AG), Pregnancies (PG), Diabetes Pedigree Function (DPF)

## Data Availability

The datasets used in the experiment are publicly available, and the URL’s are mentioned in the manuscript (Section 4). Heart Disease: https://www.kaggle.com/datasets/christofel04/cardiovascular-study-dataset-predict-heart-disea (accessed on 10 May 2026). Lung Cancer: https://www.kaggle.com/datasets/jillanisofttech/lung-cancer-detection (accessed on 10 May 2026). Diabetes: https://www.kaggle.com/code/mragpavank/pima-indians-diabetes-database (accessed on 10 May 2026). The code used in this study is openly available on GitHub (Anaconda 2.7.2 with Jupyter Notebook 7.4.5) at the following repository: https://github.com/Shahid92-Phd/MCD.git (accessed on 12 April 2026).

## Appendix A

**Table A1:** Attribute information of heart disease dataset.

Attribute	Description	Data Type	Range
Age (AG)	Age of the person in question	Years	32–70
Sex (SX)	Sex of the individual	Categorical	0–1
Smoking (SM)	If the individual is a smoker	Nominal	0 or 1
Cigarettes Per Day (CPD)	Number of cigarettes smoked per day	Categorical	0 to 70
Blood Pressure Medicine (BPM)	If the individual is taking BP tablets	Categorical	0 or 1
Prevalent Stroke (PS)	History/prevalence of stroke	Nominal	0 or 1
Prevalent Hypertension (PH)	If the individual has hypertension	Nominal	0 or 1
Diabetes (DB)	If the individual has diabetes	Nominal	0 or 1
Total Cholesterol (TC)	Cholesterol level of an individual	Continuous	107–696
Systolic Blood Pressure (SBP)	Systolic blood pressure level of the individual	Continuous	83.50–295
Diastolic Blood Pressure (DBP)	Diastolic blood pressure level of the individual	Continuous	50–142.50
Body Mass Index (BMI)	Body Mass Index value of the individual in kg/h^2^m	Continuous	15.54–56.80
Heart Rate (HR)	Heart rate of the individual	Continuous	44–143
Glucose Level (GL)	Glucose level of the individual in mg/dL	Continuous	40–394
Heart Disease (HD)	If the individual has heart disease	Binary	0 or 1

**Table A2:** Descriptive statistics of the heart disease dataset.

Attribute	Count	Mean	Std	Min	Max
AG	3390	49.56	8.57	32	70
SX		0.43	0.49	0	1
SM		0.49	0.50	0	1
CPD		8.97	11.87	0	70
BPM		0.03	0.17	0	1
PS		0.01	0.07	0	1
PH		0.31	0.46	0	1
DB		0.02	0.15	0	1
TC		237.26	44.83	107	696
SBP		132.18	21.87	83.5	295
DBP		82.89	11.77	50	142.5
BMI		25.82	4.07	15.54	56.8
HR		75.62	11.97	44	143
GL		81.67	22.11	40	394
HD		0.15	0.36	0	1

**Table A3:** Attribute information of the lung cancer dataset.

Attributes	Description	Data Type	Range
Gender (GD)	Gender of an individual	Categorical	0–1
Age (AG)	Age of an individual	Numeric	21–87
Smoking (SM)	If the individual is a smoker	Categorical	1–2
Yellow fingers (YFs)	If the individual’s fingers have turned yellow	Categorical	1–2
Anxiety (AN)	If the individual is suffering from anxiety	Categorical	1–2
Peer pressure (PP)	If the individual is influenced by peer pressure	Categorical	1–2
Chronic disease (CD)	If the individual has any chronic disease	Categorical	1–2
Fatigue (FT)	If the individual feels fatigued	Categorical	1–2
Allergy (AL)	If the individual has any allergy	Categorical	1–2
Wheezing (WH)	If the individual wheezes	Categorical	1–2
Alcohol consuming (AC)	If the individual consumes alcohol	Categorical	1–2
Coughing (CF)	If the individual coughs	Categorical	1–2
Shortness of breath (SB)	If the individual has a breathing problem	Categorical	1–2
Swallowing difficulty (SD)	If the individual experiences difficulty while swallowing	Categorical	1–2
Chest pain (CP)	If the individual has chest pain	Categorical	1–2
Lung cancer (LC)	If the individual has lung cancer	Binary	0–1

**Table A4:** Descriptive statistics of the lung cancer dataset.

Attribute	Count	Mean	Std	Min	Max
GD	309	0.52	0.50	0	1
AG		62.67	8.21	21	87
SM		1.56	0.50	1	2
YF		1.57	0.50	1	2
AN		1.50	0.50	1	2
PP		1.50	0.50	1	2
CD		1.50	0.50	1	2
FT		1.67	0.47	1	2
AL		1.56	0.50	1	2
WH		1.56	0.50	1	2
AC		1.56	0.50	1	2
CF		1.58	0.49	1	2
SB		1.64	0.48	1	2
SD		1.47	0.50	1	2
CP		1.56	0.50	1	2
LC		0.87	0.33	0	1

**Table A5:** Attribute information of the diabetes dataset.

Attribute	Description	Data Type	Range
Pregnancies (PG)	Number of times the individual is pregnant	Number	0–17
Glucose (GL)	Plasma glucose concentration	Number	0–199
Blood Pressure (BP)	Diastolic blood pressure of the individual	Number	0–122
Skin Thickness (ST)	Triceps skin fold thickness of the individual	Number	0–99
Insulin (IN)	2-Hour serum insulin of the individual	Number	0–846
Body Mass Index (BMI)	Body Mass Index of the individual in KG/m^2^	Numeric	0–67.1
Diabetes Pedigree Function (DPF)	Diabetes pedigree function of the individual	Numeric	0.08–2.42
Age (AG)	Age of the individual	Years	21–81
Diabetes (DB)	If the individual has diabetes	Binary	0–1

**Table A6:** Descriptive statistics of the diabetes dataset.

Attribute	Count	Mean	Std	Min	Max
PG	768	3.85	3.37	0	17
GL		120.89	31.97	0	199
BP		69.11	19.36	0	122
ST		20.54	15.95	0	99
IN		79.80	115.24	0	846
BMI		31.99	7.88	0	67.1
DPF		0.47	0.33	0.08	2.42
AG		33.24	11.76	21	81
DB		0.35	0.48	0	1
